# Diamine-Mediated Synergistic Engineering of Orientation and Interfacial Field of 3D/1D Heterojunctions for Efficient Perovskite Photovoltaics

**DOI:** 10.1007/s40820-026-02190-z

**Published:** 2026-04-14

**Authors:** Yaobin Li, Yunxuan Cao, Yu Zou, Wenjin Yu, Zhenhuang Su, Zhuoer Cai, Yueli Liu, Qinyun Liu, Hantao Wang, Lefan Gong, Yucheng Ye, Rong Tang, Yunan Gao, Felix Thomas Eickemeyer, Bo Qu, Lixin Xiao, Zhijian Chen

**Affiliations:** 1 State Key Laboratory for Artificial Microstructures and Mesoscopic Physics, School of Physics, Peking University, Beijing, 100871 People’s Republic of China; 2 Future Photovoltaics Research Center, Global Institute of Future Technology (GIFT), Shanghai Jiao Tong University, Shanghai, 200240 People’s Republic of China; 3 AI for Science (AI4S)-Preferred Program, Peking University Shenzhen Graduate School, Shenzhen, 518055 People’s Republic of China; 4 Laboratory of Photonics and Interfaces, Institute of Chemical Sciences and Engineering, École Polytechnique Fédérale de Lausanne (EPFL), Lausanne, Switzerland; 5 Shanghai Synchrotron Radiation Facility (SSRF), Shanghai Advanced Research Institute, Chinese Academy of Sciences, Shanghai, 201204 People’s Republic of China; 6 School of Chemistry and Chemical Engineering, Southeast University, Nanjing, 211189 People’s Republic of China

**Keywords:** Perovskite, Solar cell, 3D/1D heterojunction, Oriented growth, Field effect passivation

## Abstract

The performance of three-dimensional (3D) perovskite solar cells (PSCs) is predominantly limited by interfacial non-radiative recombination and instability. Although low-dimensional (LD) interlayers, particularly two-dimensional (2D) perovskites, are widely adopted for surface passivation, their heterogeneous n-values and quantum-well confinement often impede charge transport. One-dimensional (1D) perovskites offer a promising alternative due to their structural flexibility and superior passivation capabilities, yet their potential has been underexploited by challenges in controlled crystallization and ordered orientation. Here, we constructed a 3D/PDAI_2_/1D heterojunction through sequential deposition of propane-1,3-diammonium iodide (PDAI_2_) and 4-amidinopyridinium chloride (4APyCl). The pre-anchored PDAI_2_ not only provides field-effect passivation but also templates the subsequent vertical alignment of 1D Pb–I chains assembled with 4APyCl. This configuration establishes continuous out-of-plane charge transport channels, enabling effective surface defect passivation, favorable energy-level alignment, and enhanced interfacial carrier extraction. The resulting inverted PSCs achieved a champion power conversion efficiency of 25.8% and retained 85% of the initial performance after 1000 h of maximum power point tracking under 1-sun illumination. By demonstrating the critical role of molecular orchestration in LD interlayers, this work provides a blueprint for establishing structure–property relationships and guides the rational design of stable and efficient 3D/1D perovskite photovoltaics.

**Supplementary Information:**

The online version contains supplementary material available at 10.1007/s40820-026-02190-z.

## Highlights

- Constructing 3D/PDAI_2_/1D heterojunction through sequential deposition of propane-1,3-diammonium iodide (PDAI_2_) and 4APyCl.
- The pre-anchored PDAI_2_ not only provides field-effect passivation but also template the subsequent vertical alignment of 1D Pb–I octahedral chains featuring continuous out-of-plane charge transport channels.
- Perovskite solar cells containing 3D/1D structure deliver a champion power conversion efficiency of 25.8% and retain 85% of their initial efficiency after 1000 h maximum power point tracking in accordance with the ISOS-L-1 protocol.

**Supplementary Information:**

The online version contains supplementary material available at 10.1007/s40820-026-02190-z.

## Graphical abstract

**Figure:**
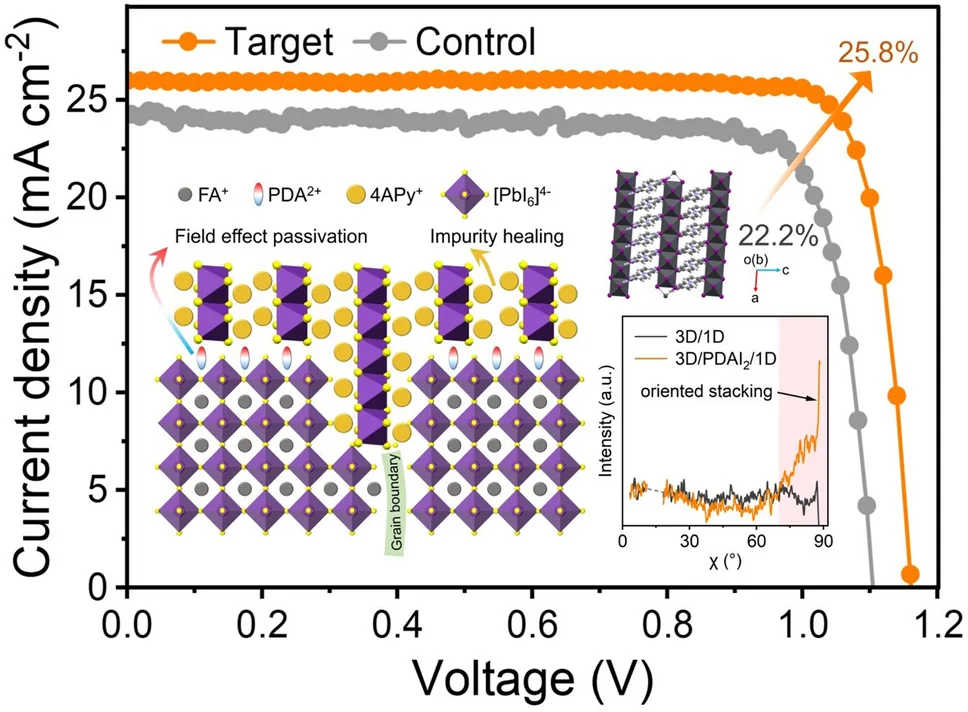

## Introduction

The photoelectric conversion efficiency (PCE) of three-dimensional (3D) perovskite solar cells (PSCs) has risen from 3.8% in 2008 to nearly 27% currently [1, 2], indicating the significant application prospects of perovskite in the photovoltaic area [3]. In PSCs, the interface between the active layer and the charge transport layer, owing to factors such as numerous defects, lattice mismatch, thermal expansion coefficient mismatch, and energy-level mismatch, can result in severe non-radiative recombination or ion migration, which compromises the device efficiency and stability [4, 5].

Low-dimensional (LD) interfacial layers are extensively employed to passivate surface and grain boundary defects, suppress non-radiative recombination, facilitate energy band matching, and enhance the efficiency and stability of devices [6–9]. Typically, during the solution processing step, the two-dimensional (2D) perovskite layer is formed by spin-coating 2D organic ligands to the surface of 3D perovskite and annealing [10, 11]. A key challenge in practical fabrication is the prevalent formation of mixed 2D perovskite phases, which exhibit discrete n-values and orientations that severely restrict charge transport. This variability imposes stringent demands on achieving controlled crystallization and reproducible processing of 2D perovskites [12]. Further compounding the issue, the discrete [PbI_6_]^4−^ layered framework gives rise to an unfavorable quantum-well structure, thereby limiting the efficiency of interfacial carrier transfer [13–15].

By contrast, one-dimensional (1D) perovskites featuring wire-like Pb–I frameworks exhibit greater structural flexibility and variability, alongside superior passivation capabilities at defect sites like grain boundaries [16–18]. More importantly, unlike the planar 2D counterparts that restrict carrier transport, the spatially more flexible 1D frameworks enable the construction of continuous charge extraction channels, especially when the Pb–I chains are oriented out of plane, thereby optimizing transport efficiency [16, 19]. The advancement of post-solvent rinsing methods has enabled precise orientation control of low-dimensional perovskites in 3D/2D heterojunctions [13, 14]. Nevertheless, a systematic understanding of how to direct crystallization for achieving uniform and charge-transport-optimized orientations in 1D perovskites remains elusive [20]. Consequently, developing strategies to achieve uniform out-of-plane orientation in 1D perovskites is crucial for harnessing their intrinsic advantages, yet this pivotal bottleneck has often been overlooked, hindering further progress in 3D/1D structured perovskite photovoltaics. Fine-tuning of post-treatment molecular characteristics significantly governs the optoelectronic properties of passivation layers [21]. Previous studies demonstrated that a chlorine-rich environment with suitable cations can promote 1D perovskite formation [22–26]. And organic amidinium cations featuring resonance-enhanced N–H bonds can resist deprotonation and suppress lateral energy landscape inhomogeneity [27–29]. These findings inspired the further design of post-treatment strategies and molecular-level modulation.

Herein, we propose a diamine-mediated synergistic strategy to investigate the influence of diamine ligands in the assembly of 1D perovskites derived from 4APyCl. Upon sequential deposition of propane-1,3-diammonium iodide (PDAI_2_) and 4-amidinopyridinium chloride (4APyCl), the pre-deposited PDAI_2_ anchors at A-site cation vacancies on the surface while simultaneously serving as field-effect passivation. Subsequently deposited 4APyCl further assembles with residual components such as free PbI_2_ to form 1D perovskites. Notably, in contrast to the randomly oriented structures obtained by direct deposition of 4APyCl, the initially adsorbed PDAI_2_ guides the vertical alignment of Pb–I octahedral chains and promotes their ordered assembly with 4APyCl to form 1D perovskites, establishing continuous out-of-plane carrier transport channels. Consequently, the integrated 3D/PDAI_2_/1D heterojunction exhibits effective healing of surface impurity and defects, along with optimized interfacial electric fields and energy-level alignment, as well as efficient 1D charge transport channels. These synergistic improvements collectively suppress non-radiative recombination losses and facilitate enhanced interfacial carrier extraction. The resulting inverted device achieved a champion PCE of 25.8%, ranking it among the top-performing 3D/1D structured perovskite solar cells. Furthermore, reduced impurity and the conformal 1D interfacial capping layer contribute to superior operational stability, enabling unencapsulated devices to retain 85% of their initial PCE after 1000-h operation of continuous 1-sun illumination following the ISOS-L-1 protocol.

## Experimental Section

### Materials

*N*,*N*-Dimethylformamide (DMF, 99.8%), dimethyl sulfoxide (DMSO, 99.9%), methanol (99.9%), ethanol (99.5%), isopropanol (IPA, 99.5%), and chlorobenzene (CB, 99.8%) were purchased from Sigma-Aldrich. [6,6]-Phenyl-C61-butyric acid methyl ester (PCBM, 99.9%) was purchased from Advanced Election Technology Co. [4-(3,6-Dimethyl-9*H*-carbazol-9-yl)butyl]phosphonic acid (Me-4PACz, > 98.0%), 2-(9*H*-carbazol-9-yl)ethyl)phosphonic acid (2PACz, > 98.0%), and formamidinium iodide (FAI, 99.99%) were purchased from TCI. Methylammonium chloride (MACl, 99.9%), cesium iodide (CsI, 99.999%), lead iodide (PbI_2_, 99.99%), lead bromide (PbBr_2_, 99.99%), 1,3-propyldiammonium diiodide (PDAI_2_, 99.5%), and bathocuproine (BCP, 99%) were purchased from Xi’an Polymer Light Technology Corporation. Methylammonium iodide (MAI, 99.99%) was purchased from Greatcell Solar Materials. 4-Amidinopyridinium chloride (4APyCl, 98%) was purchased from Innochem. All these commercially available materials were used as received without further purification.

### Synthesis of 1D Perovskite (4APy)_2_PbI_4_

157.6 mg of 4APyCl and 461 mg of PbI_2_ were dissolved in 4 mL of DMF. The solution was stirred and dissolved at a temperature of 100 °C or higher, followed by cooling at a rate of 5 °C h^−1^. This process yielded white needle-like 1D single crystals at the bottom of the vial.

### Device Fabrication

The ITO substrates were ultrasonically cleaned with water, acetone, and IPA for 30 min, respectively. After UV-light treatment for 20 min, the ITO substrates were transferred to N_2_ glove box. The Me-4PACz solution (0.33 mg mL^−1^ dissolved in ethanol) was spin-coated on the ITO substrates at 3000 rpm for 30 s and annealed at 100 °C for 10 min. 1.4 M perovskite precursor solution was prepared by adding FAI, PbI_2_, CsI, MAI, MACl, and PbBr_2_ into a mixed DMF/DMSO solvent with the ratio of 4:1 following the formula of Cs_0.05_(FA_0.95_MA_0.05_)_0.95_Pb(I_0.98_Br_0.02_)_3_ and stirred until dissolved. Perovskite solutions were deposited by a two-step spin-coating of 1000 rpm for 10 s and 5000 rpm for 30 s. At 15 s before the end of the procedure, 200 μL CB was cast onto the film and annealed at 100 °C for 20 min. For the fabrication of 3D/PDAI_2_/1D, the PDAI_2_ solution (0.5 mg mL^−1^ dissolved in a 1:1 mixed solvent of CB and IPA) was spin-coated on the 3D films at 5000 rpm for 30 s and annealed at 100 °C for 5 min. Afterward, the 4APyCl solution (1 mg mL^−1^ dissolved in IPA) was spin-coated on the 3D/PDAI_2_ substrates at 5000 rpm for 30 s and annealed at 80 °C for 5 min. Then, the PCBM solution (20 mg/ml dissolved in CB) was spin-coated at 1500 rpm for 30 sand annealed at 70 °C for 10 min. The saturated supernatant of BCP (dissolved in methanol) was spin-coated at 6000 rpm for 30 s, followed by annealing at 70 °C for 5 min. Finally, 100 nm thickness of Ag was thermally evaporated as an electrode using a shadow mask with aperture area of 0.0672 cm^2^. For devices fabricated on FTO substrates to achieve further efficiency improvement, the ITO substrates were replaced with FTO substrates with anti‑reflective coatings, and the solutions of HTL layer were replaced with a mixture of Me‑4PACz and 2PACz (0.33 mg mL^−1^ and 0.17 mg mL^−1^ in ethanol). All other materials and deposition methods remained unchanged.

### Characterization

The SEM images were characterized by Hitachi S-4800. The XRD patterns were performed by powder X-ray diffractometer (PANalytical) using Cu Kα radiation (*λ* = 1.5418 Å) at 40 kV and 40 mA. The XPS spectra and UPS spectra were characterized by Thermo Scientific Nexsa (voltage: 12000.00 V, current: 6 mA, vacuum: *P* < 10–9 mBar, work FN: 4.20 eV). The steady-state PL and TRPL spectra (excitation at 450 nm) were characterized by FLS1000 (Edinburgh Instruments). The FTIR measurement was characterized by Nicolet iS50 (Thermo Fisher Scientific). The simulated AM 1.5G illumination (100 mW cm^−2^) sunlight was provided by Newport Thermal Oriel 69911300W solar simulator. Photovoltaic performances of PSCs were measured by Keithley 2611 source meter. GIWAXS was conducted by the BL14B1 beamline at Shanghai Synchrotron Radiation Facility (SSRF). The corresponding X-ray beam possesses a wavelength of 1.24 Å at a grazing incidence angle of 0.1° and uses an energy of 10 keV. The instruments and parameters used for the measurement and calibration of PLQY are described in the literature [30]. QFLS is derived from PLQY using the following formula: QFLS = *qV*_OC, SQ_ + *k*_B_*T*ln(PLQY), where q is the elementary charge, *V*_OC, SQ_ is the Shockley–Queisser limit of the open-circuit voltage for the corresponding bandgap, *k*_B_ is the Boltzmann constant, and *T* = 298.15 K, which is the sample temperature [31]. The AFM and KPFM measurements were characterized by Cypher ES (Oxford Instruments). While measuring each sample, we also measured the potential of a standard sample (ITO). Based on the potential difference and the work function of the standard sample, the surface work function was determined. The EQE spectra were obtained using a QE-R 3011 system (Enlitech, Taiwan) in the range of 300–900 nm. The Mott–Schottky test was characterized by electrochemical workstation (CHI760E, CH Instruments, Inc.) Maximum power point tracking (MPPT) was obtained using a multi-channel solar cell and module stability testing system (MSCLT-1, 91PVKSOLAR).

### Calculation

The Gaussian electrostatic potential of the 4APyCl was calculated in the Gaussian 09 package at the B3LYP/def2TZVP level with DFT-D3 [32].

## Results and Discussion

### Fabrication of 3D/PDAI_2_/1D Heterojunction

**Fig. 1:**
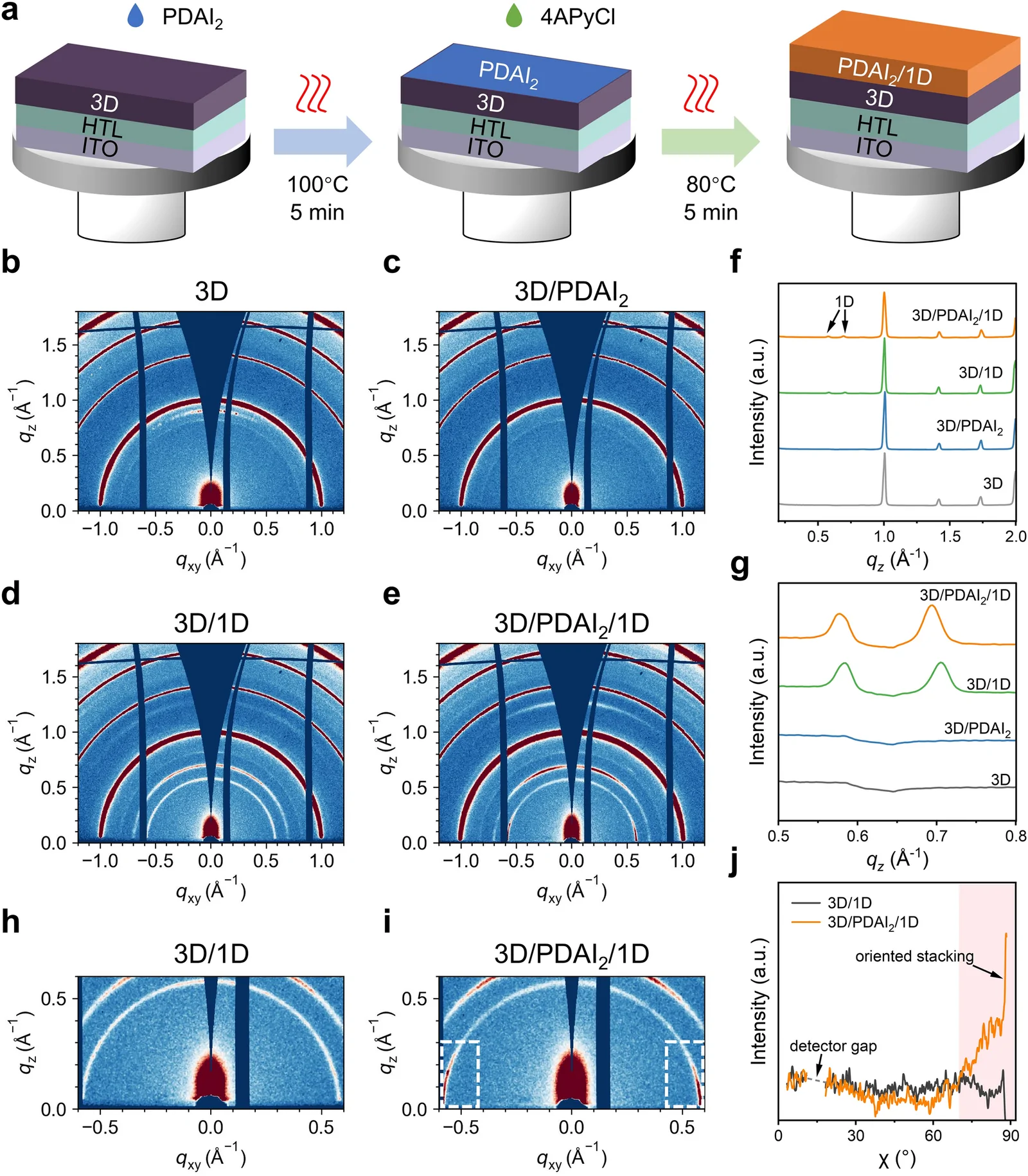
The fabrication of 3D/PDAI_2_/1D heterojunction. **a** Process flow diagram of the fabricating of 3D/PDAI_2_/1D heterojunction. GIWAXS images of **b** 3D, **c** 3D/PDAI_2_, **d** 3D/1D, and **e** 3D/PDAI_2_/1D structured perovskite films. **f**–**g** The GIWAXS intensity profiles near the *q*_*z*_ direction for different samples. Zoom-in view of the GIWAXS patterns near the diffraction ring at *q* = 0.58 Å^−1^ of **h** 3D/1D and **i** 3D/PDAI_2_/1D structured films. **j** Intensity–*χ* plot of the diffraction ring at *q* = 0.58 Å^−1^

For the preparation of the 3D/PDAI_2_/1D heterojunction (Fig. 1a), 3D perovskite was initially fabricated on ITO/SAM via a one-step anti-solvent approach. Subsequently, PDAI_2_ was deposited to form a field-effect passivation layer. The molecule features ammonium groups at both ends: One binds to A-site cation vacancies on the surface, and the other induces a surface dipole field that repels holes near the interface [32–35]. Following the spin-coating of 4APyCl, conduct annealing to form a low-dimensional capping layer. The chemical structure of PDAI_2_ and 4APyCl is presented in Fig. S1. The molecular structure of 4APyCl exhibits a D-A dipole configuration (Fig. S2). The positive charge is predominantly concentrated on the amidine group, whereas the negative charge is mainly localized on the N atom of the pyridine. Synchrotron radiation accelerator-based grazing incidence wide-angle X-ray scattering analysis (GIWAXS) was performed to investigate the impact of the post-treatment of 3D perovskite surfaces [36]. By setting the grazing incidence angle at 0.1°, we were able to acquire the surface information of the post-treated films (Fig. 1b–e). Figure 1f–g presents the out-of-plane (*q*_*z*_) integration of GIWAXS patterns. On the surface of the 3D perovskite film, a signal of excess PbI_2_ can be detected at *q* ~ 0.9 Å^−1^ [37, 38]. Nevertheless, treatment with either PDAI_2_ or 4APyCl can cause the signal of excess PbI_2_ to vanish. These two passivating molecules bind to and remove excess PbI_2_ impurities, which are known to be detrimental to the stability of perovskite solar cells [39, 40].

**Fig. 2:**
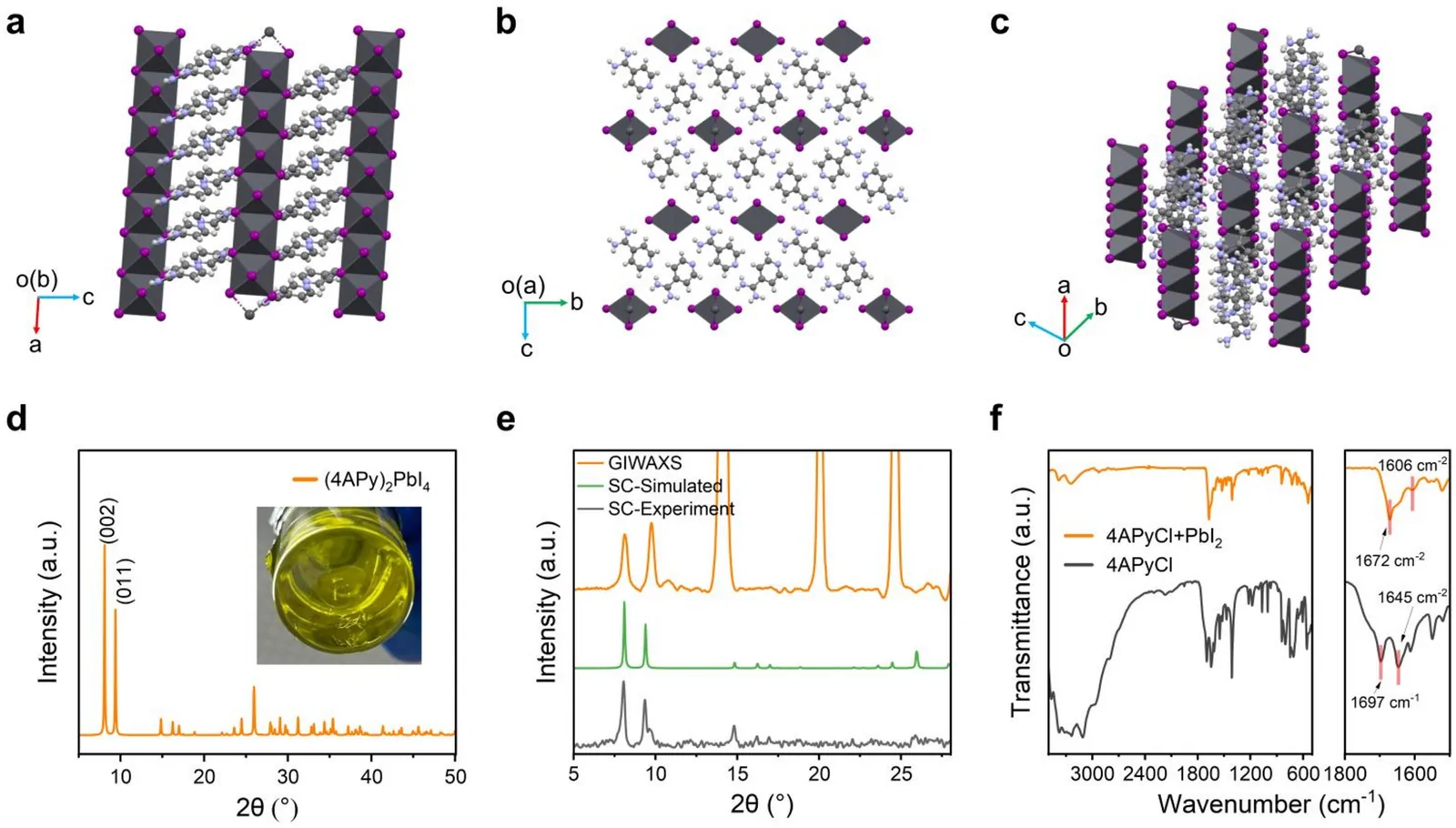
Crystal structure of 1D perovskite (4APy)_2_PbI_4_. **a**–**c** Crystallographic packing diagrams of 1D (4APy)_2_PbI_4_ perovskites when viewed along different directions. **d** single-crystal XRD patterns for (4APy)_2_PbI_4_. (Inset) Photograph of a (4APy)_2_PbI_4_ single crystal. **e** Comparison of experimental and simulated powder XRD patterns for (4APy)_2_PbI_4_ perovskites, and the GIWAXS intensity profile near the *q*_*z*_ direction for the 3D/PDAI_2_/1D structured film. **f** FTIR spectra of 4APyCl and the mixture of 4APyCl and PbI_2_

After PDAI_2_ treatment, no new diffraction rings at lower angles were detected, indicating the absence of low-dimensional structures (Figs. S3 and S4), which is consistent with the findings in the previous literature [33]. However, after treatment with 4APyCl, diffraction rings are observed at *q* = 0.58 Å^−1^ and *q* = 0.69 Å^−1^ (Fig. 1d–i). We initially hypothesize that these two inner rings are low-dimensional perovskites formed by the reaction of 4APyCl with PbI_2_ (Fig. S5), corresponding specifically to the (002) and (011) crystal planes of a 1D perovskite, as detailed in the next section (Fig. 2). Based on previous research, compared to I⁻ anions, the smaller Cl⁻ anions further stabilize the 1D phase by increasing its formation energy [23]. We replaced Cl⁻ anions with I⁻ anions and investigated the XRD patterns of the products from the reaction between 4APyI and PbI_2_, as shown in Fig. S6. The reaction of 4APyI with PbI_2_ may yields a two-dimensional perovskite, which requires further investigation.

The analysis of the angular dependence of intensity in GIWAXS patterns (intensity-*χ*) enables the identification of spatial orientation characteristics in crystal structures of 1D layer, such as preferential or random orientation [41]. In contrast to the randomly oriented 1D structures obtained through direct deposition of 4APyCl, the 1D structures fabricated via sequential deposition of PDAI_2_/4APyCl exhibit ordered orientation (Fig. 1h–i). The diffraction ring of 1D structures at *q* = 0.58 Å^−1^ demonstrated concentrated high intensity near *χ* ≈ ± 90° (as indicated within the white frame, Fig. 1i), confirming a unified preferential in-plane orientation of the 1D perovskite (Fig. 1j), where the Pb–I chains predominantly align perpendicular to the surface of the 3D film along the out-of-plane direction. We propose that, consistent with prior reports [27], the initially deposited PDAI_2_ molecules insert and anchor at the A-sites of 3D perovskite surface with their distal ends exposed outward, thereby pre-occupying out-of-plane binding sites. This steric hindrance governs the subsequent binding mode of 4APyCl to the Pb–I framework, guiding its predominantly in‑plane alignment and insertion to form bonds that construct vertical Pb‑I chains. Consequently, highly oriented 1D perovskite are constructed, featuring Pb–I chains perpendicular to the 3D perovskite surface. These out-of-plane inorganic chains, together with the field-effect passivation by the pre-deposited PDAI_2_, establish a multifunctional surface modulation layer.

Consistent with the elimination of the PbI_2_ ring in GIWAXS patterns, the scanning electron microscope (SEM) results revealed that the treatment with PDAI_2_ and 4APyCl effectively removes the excess PbI_2_ (manifested as bright area) on the surface of the 3D perovskite (Fig. S7) [42]. Notably, after the 4APyCl treatment, the grains exhibit flatter and neater (Fig. S8), larger grain size (Fig. S9), and enhanced crystallinity. Among the conditions investigated, the 3D/PDAI_2_/1D sample demonstrates the largest average grain size.

### Crystal Structure of 1D Perovskite (4APy)_2_PbI_4_

To further verify the crystal structure of the 1D perovskites, single crystals were synthesized from a solution of 4APyCl and PbI_2_ in DMF using the solution cooling method [22, 43]. The lattice structure was analyzed using single-crystal X-ray diffraction (SCXRD), as depicted in Fig. 2a–c, and the crystal structure parameters are presented in Tables S1 and S2 (CCDC number: 2512007). The edge-shared inorganic Pb–I octahedral chains extend along the *a* direction. In the *b* and *c* directions, large-sized organic cation ligands separate the one-dimensional Pb–I octahedral chains. Accordingly, the grown single crystals are 1D rod-like (Fig. 2d inset). The single-crystal XRD patterns confirms (Fig. 2d) that the Miller indices of the peaks around 8.1° and 9.4° are (002) and (011). We transformed the abscissa *q*_*z*_ of the out-of-plane integration of the GIWAXS pattern of 3D/PDAI_2_/1D sample (Fig. 1e) into 2*θ* and compared it with the experimental and simulated XRD patterns of the one-dimensional perovskite 4APy_2_PbI_4_ (Fig. 2e). The two peaks in the range of 5°–10° can match well. Therefore, it can be confirmed that when 3D perovskite undergoes post-treatment with 4APyCl, one-dimensional perovskite forms on its surface. Compared to DMF, the use of HI solvent to dissolve PbI_2_ and 4APyCl for single-crystal growth yielded larger and higher-quality crystals. The orange crystals obtained correspond to the 2D perovskite 4APyPbI_4_, and their XRD results do not match the GIWAXS data of the 3D/4APyCl sample (Figs. S10 and 1d, Note S1). The (002) plane of the 1D perovskite on the surface of 3D/PDAI_2_ exhibits a preferential in-plane orientation, whereas the (002) plane of the 1D perovskite on the surface of 3D shows a random orientation. PDAI_2_ guides the vertical alignment of Pb–I octahedral chains and promotes their ordered assembly with 4APyCl to form 1D perovskites, establishing continuous out-of-plane carrier transport channels. Consequently, the carriers are not impeded by organic molecules with low conductivity.

Fourier transform infrared spectroscopy (FTIR) results (Fig. 2f) revealed that after mixing with PbI_2_, the C=N stretching vibration peak of the FA group of 4APyCl shifts from 1697 to 1672 cm^−1^, and the C=N stretching vibration peak of the pyridine moiety shifts from 1645 to 1606 cm^−1^ [44]. We further evaluated the photoelectric characteristics (Fig. S11) of (4APy)_2_PbI_4_, which showed a photoluminescence (PL) peak at 538 nm, an optical bandgap of 2.77 eV, and a work function of 4.57 eV. Although one-dimensional perovskites exhibit a broader bandgap, the thin-surface passivation layer does not modify the optical bandgap of the perovskite light-absorbing layer (Fig. S12). Additionally, photoelectrical characterizations confirmed the stability of the 1D perovskite polycrystalline thin films in air (Fig. S13).

### Energy-Level Structure of Perovskite Surfaces under Different Passivation Conditions

**Fig. 3:**
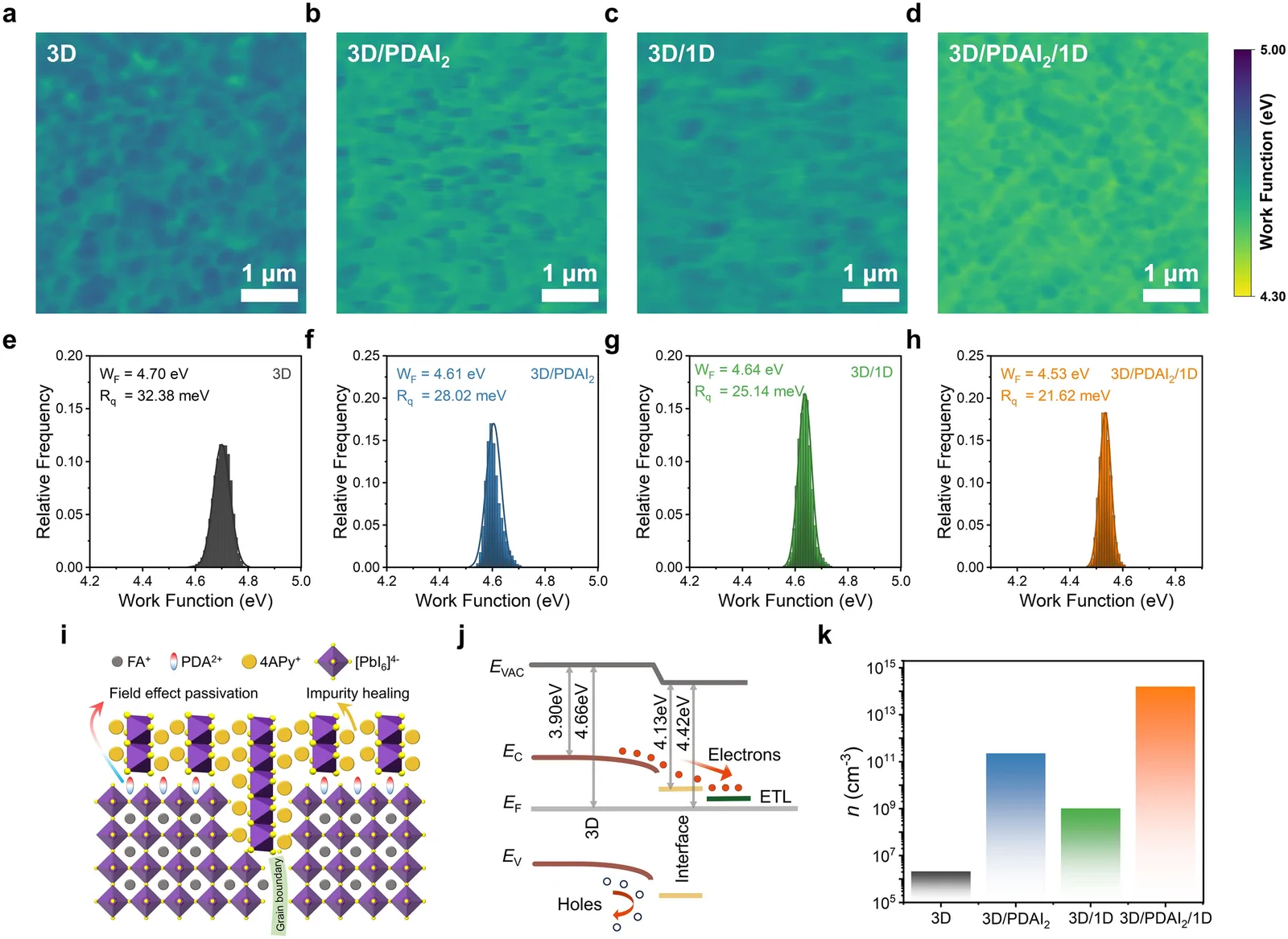
Energy-level structure of perovskite surfaces under different passivation conditions. KPFM images of **a** 3D, **b** 3D/PDAI_2_, **c** 3D/1D, and **d** 3D/PDAI_2_/1D perovskite surfaces. **e**–**h** Statistical distribution of surface work function. **i** Schematic diagram of the surface structure of 3D/PDAI_2_/1D. **j** Schematic diagram of the energy-level structure of 3D/PDAI_2_/1D heterojunctions. **k** Calculated concentration of electrons in the conduction band

Kelvin probe force microscopy (KPFM) measurements were employed to characterize the electrical properties of the surfaces of perovskite with different post-treatments (Fig. 3a–d). Consequently, the distribution information of the surface potential and surface work function of the films, along with their average values and the root mean square (*R*_*q*_), was obtained (Fig. 3e–h). The surface of the 3D perovskite had an average work function of 4.70 eV. After being modified with PDAI_2_ on 3D films solely, its work function decreased to 4.61 eV, which could be attributed to the field-effect passivation of PDAI_2_ [32]. After modification with 1D on 3D films solely, its work function decreased to 4.64 eV, owing to the electron-donating pyridine groups that increased the surface electron concentration [45]. Under the synergistic effect of the PDAI_2_ and 1D passivation, the surface work function decreased to 4.53 eV, with the smallest *R*_*q*_ value of 21.62 meV, indicating the best uniformity of the surface potential. Previous research has indicated that a uniform surface potential is advantageous for enhancing the fill factor (FF) of devices [46]. By measuring the energy band structures of various film surfaces using ultraviolet photoelectron spectroscopy (UPS) (Fig. S14), we were able to obtain the energy band structure diagrams (with the vacuum level unified) (Fig. S15). As depicted in Fig. 3i, the dipole field introduced by PDAI_2_ modification coincides with the direction of the built-in electric field in the 3D/electron transport layer (ETL), thus enhancing it and facilitating electron transport. Concurrently, excess PbI_2_ impurities are converted into a 1D perovskite capping layer, which exhibits a homogenized morphology and uniform surface potential. The synergistic n-doping effect of PDAI_2_ and the 1D perovskite optimizes the energy-level alignment between the modified 3D perovskite and the ETL, thereby collectively facilitating electron extraction and suppressing hole transfer (Fig. 3j).

3D/PDAI_2_/1D exhibits the smallest difference between the conduction band energy level (*E*_C_) and the Fermi energy level (*E*_F_). According to Eq. (1) [47]:

1 $$n={N}_{\mathrm{C}}\mathrm{exp}\left(-\frac{{E}_{\mathrm{C}}-{E}_{\mathrm{F}}}{{k}_{\mathrm{B}}T}\right)$$

where *N*_C_ is the effective density of states in the conduction band, *k*_B_ is the Boltzmann constant, and *T* is the thermodynamic temperature. Through calculation, it can be obtained that the 3D/PDAI_2_/1D has the highest surface electron concentration of 1.65 × 10^14^ cm^−3^ (Figs. S16 and 3k).

### Photoelectric Properties of Perovskite under Different Post-Passivation Strategies

**Fig. 4:**
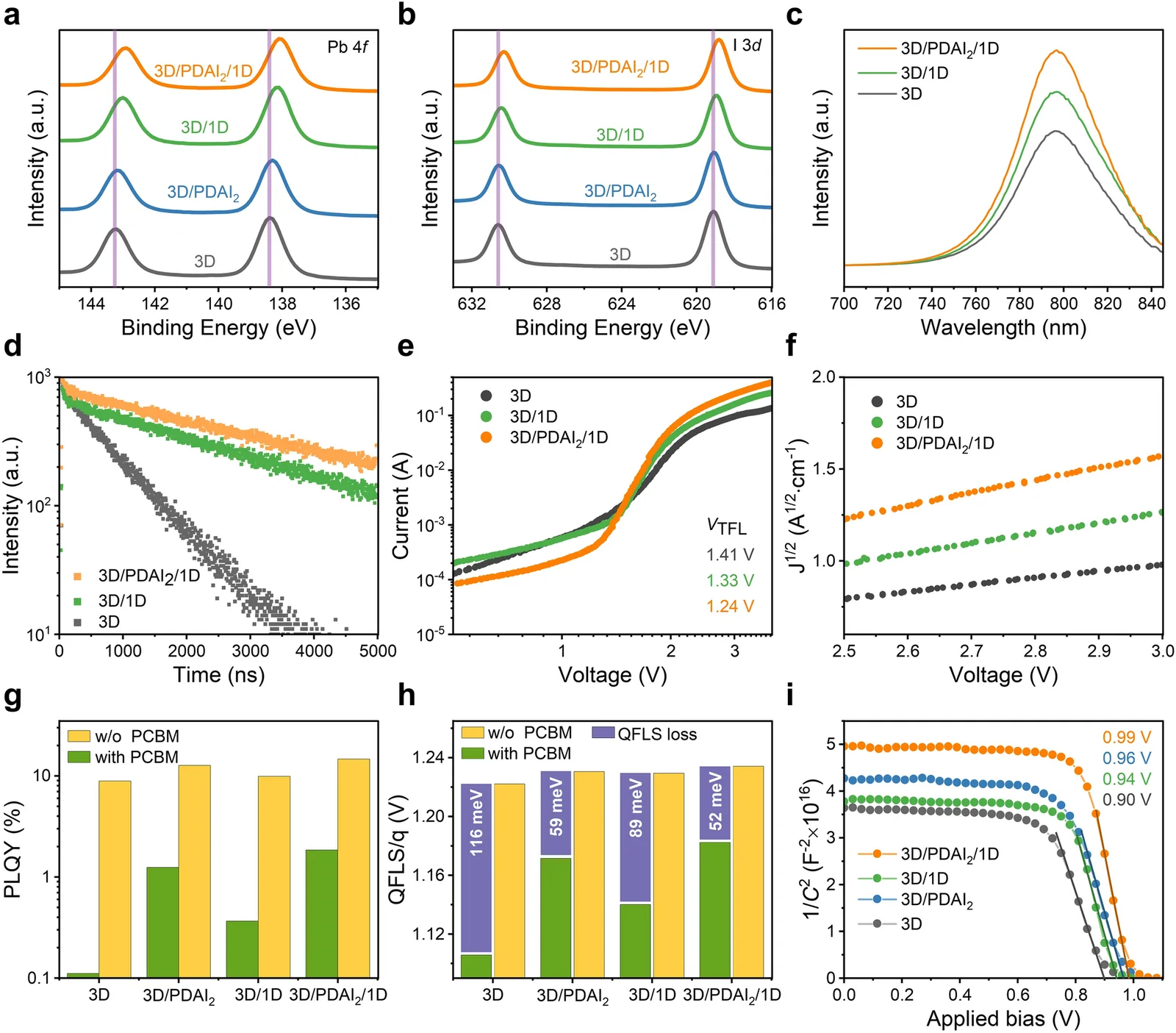
Photoelectric properties of perovskite under different post-passivation strategies. **a** Pb 4*f* and **b** I 3*d* XPS spectra of different perovskite films. **c** Steady-state PL spectra and **d** TRPL spectra of different perovskite films. **e** SCLC plots for single-carrier devices based on the different structured perovskites. **f** *J*^1/2^–*V* curves in the child region of the single-carrier devices. **g** PLQY and **h** QFLS results of perovskite films with different structures, with and without the incorporation of PCBM as the ETL. **i** Mott–Schottky plots of PSCs with different structures

X-ray photoelectron spectroscopy (XPS) was used to characterize the interactions between passivation molecules and 3D perovskites (Fig. 4a, b). When PDAI₂ was deposited solely on 3D perovskite, there were no significant shifts in the Pb 4*f* peak and I 3*d* peak. However, after depositing the 1D passivation layer, it could be observed that both the Pb 4*f* and I 3*d* peaks shifted toward lower binding energies. The N on the pyridine ring of 4APyCl acts as an electron donor, increasing the electron density around the Pb and I atoms, enabling the uncoordinated Pb^2+^ defects to be well passivated. Effective defect passivation enables the 3D/1D film to exhibit higher steady-state photoluminescence (PL) intensity and a longer carrier lifetime compared to the 3D film (Fig. 4c, d). The 3D/PDAI_2_/1D film, with a more ordered 1D perovskite orientation achieved through the mediation of PDAI_2_, can more effectively suppress non-radiative recombination [48]. As a result, it demonstrates the highest PL intensity and the longest carrier lifetime (Table S3). Next, single-electron-carrier devices of perovskite with different structures were constructed to calculate the electron trap state density (*n*_trap_). According to the space-charge-limited current (SCLC) method, we can extract the trap-filled limit voltage (*V*_TFL_) from the dark *J*–*V* curves of these devices (Fig. 4e), and it is known to satisfy the following Eq. (2):

2 $${n}_{\mathrm{trap}}=\frac{2{\varepsilon }_{0}{\varepsilon }_{\mathrm{r}}{V}_{\mathrm{TFL}}}{e{L}^{2}}$$

where *e* is the elementary charge, *ε*_0_ is vacuum dielectric constant, *ε*_r_ is relative dielectric constant of perovskite, and *L* is the thickness of the perovskite film. Based on this formula, the *n*_trap_ of the 3D/PDAI_2_/1D film is calculated to be 1.79 × 10^16^ cm^−3^, which is lower than 2.03 × 10^16^ cm^−3^ of the 3D film and 1.92 × 10^16^ cm^−3^ of the 3D/1D film. For single-electron-carrier devices, the relationship between *J* and *V* satisfies Eq. (3) [49]:

3 $$J=\frac{9}{8}{\varepsilon }_{0}{\varepsilon }_{\mathrm{r}}\mu \frac{{V}^{2}}{{L}^{3}}$$

where *J* is current density, *μ* is carrier mobility, and *V* is voltage applied on devices. By fitting the *J*^1/2^–*V* curves in the child region of the single-carrier devices (Fig. 4f), the electron mobility of the 3D/PDAI_2_/1D film can be obtained as 2.18 × 10^–2^ cm^2^ V^−1^ s^−1^, which is higher than that of the 3D film (6.65 × 10^–3^ cm^2^ V^−1^ s^−1^) and the 3D/1D film (1.53 × 10^–2^ cm^2^ V^−1^ s^−1^).

Furthermore, photoluminescence quantum yield (PLQY) and corresponding quasi-Fermi-level splitting (QFLS) were employed to investigate the mechanisms suppressing defect-assisted carrier recombination losses in the novel heterostructure (Fig. 4g, h). In the absence of the PCBM ETL, the deposition of PDAI_2_ and the 1D passivation layer enhanced the QFLS of the perovskite heterojunction compared to bare 3D films, owing to effective passivation of surface impurities by the post-treatment [50, 51]. The 3D/PDAI_2_/1D film achieved the highest peak PLQY of 14.67% and a QFLS of 1.234 eV, which is attributed to sufficient chemical passivation enabled by the ordered coverage of the PDAI_2_-mediated 1D perovskites. After PCBM deposition, additional interfacial recombination at the perovskite/ETL interface resulted in a reduction in both PLQY and QFLS [52–54]. Notably, the 3D/PDAI_2_/1D film exhibited the smallest QFLS loss (52 meV) upon PCBM deposition. This can be ascribed not only to the field-effect passivation capability of PDAI_2_ itself, but also to the ordered 1D perovskite channels that promotes efficient carrier separation and transport at the interface [55]. These two effects act synergistically to suppress non-radiative recombination during interfacial charge transfer (Fig. 4g, h). Therefore, the 3D/PDAI_2_/1D heterostructure ensures highly efficient carrier transport throughout the entire device, significantly enhancing its performance potential. By performing Mott–Schottky tests on complete solar cell devices, the curves of 1/*C*^2^ and applied bias voltage *V* can be obtained (Fig. 4i) and fitted by Eq. (4):

4 $$\frac{1}{{C}^{2}}=\frac{2\left({V}_{\mathrm{bi}}-V\right)}{{A}^{2}e{\varepsilon }_{0}{\varepsilon }_{\mathrm{r}}}$$

where *A* is the area of the device. The device with the 3D/PDAI_2_/1D structure has a built-in electric field of 0.99 V, which is higher than 0.90 V of the 3D device, 0.96 V of the 3D/PDAI_2_ device, and 0.94 V of the 3D/1D device. A higher built-in electric field is beneficial for improving the open-circuit voltage (*V*_OC_) of the device [56, 57].

### Performance of PSCs Containing Different Structures and Their Stability

**Fig. 5:**
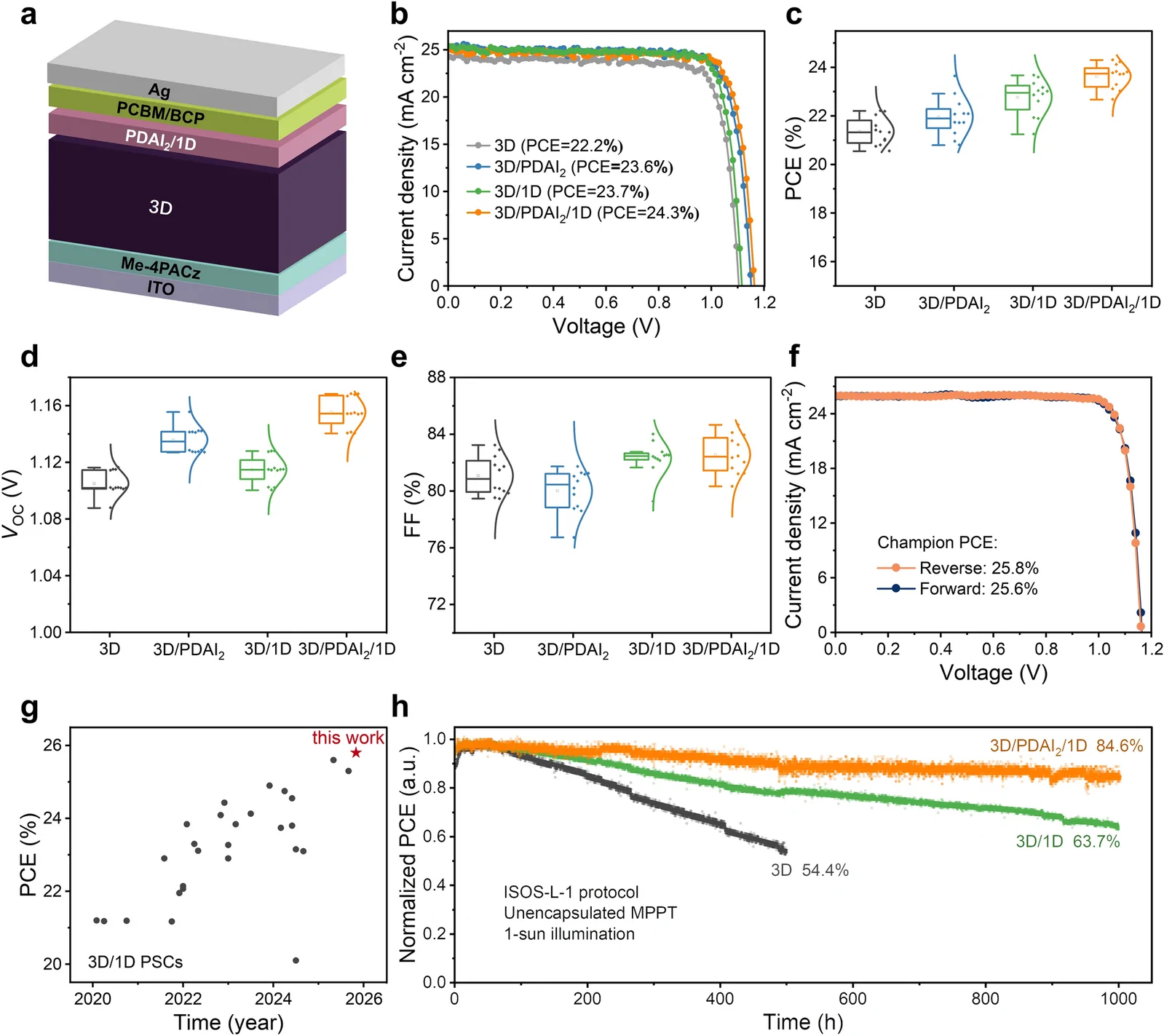
Performance of PSCs containing different structures and their stability. **a** Diagram of device structure. **b** *J*–*V* characteristics of the best-performing PSCs with different structure based on the ITO substrates without anti-reflective layer. Box plot of **c** PCE, **d** *V*_OC_, and **e** FF of PSCs with different structures. **f ***J*–*V* characteristics of the best-performing 3D/PDAI_2_/1D structured PSCs based on the FTO substrates with an anti-reflective layer. **g** Summary of recently reported PSCs based on 3D/1D heterojunctions (PCE > 20%). **h** MPPT of PSCs under continuous 1-sun illumination according to ISOS-L-1 protocol

We fabricated PSCs with an inverted structure: ITO/[4-(3,6-Dimethyl-9*H*-carbazol-9-yl)butyl]phosphonic acid (Me-4PACz)/3D/post-treatment layer/PCBM/BCP/Ag (Figs. 5a and S17), and the *J*–*V* characteristics of the best-performing PSCs with different post-treatments are shown in Fig. 5b. We found that compared to control PSCs, the 3D/PDAI_2_/1D structure led to greatest efficiency improvement (24.3% compared with 22.2%). This can be ascribed to the collective improvement in the *V*_OC_ (1.102 to 1.167 V), short-circuit current density (*J*_SC_, from 24.3 to 25.0 mA cm^−2^), and fill factor (FF) (from 82.9% to 83.3%) (Fig. S18, Table S4) and well in agreement with the EQE spectra and stabilized power output (SPO) data (Figs. S19 and S20). Statistic photovoltaic parameters are summarized in Fig. 5c–e, which demonstrate reproducibility of the performance improvement of PSCs with different post-treatments. *V*_OC_ increments stem from the improved QFLS and *V*_bi_ due to the synergistic n-doping of PDAI_2_ and 4APyCl, which precisely regulate surface energy levels and reduce interfacial recombination. Meanwhile, the morphological and potential homogenization achieved through 1D passivation contributes to the improvement of FF. At low concentrations, the post-treatment exhibits a relatively weak passivation effect, leading to limited enhancement in *V*_OC_. Conversely, higher concentrations impair the device’s *J*_SC_ and FF. Therefore, for the 3D/PDAI_2_/1D device, the concentration of PDAI_2_ was optimized to 0.5 mg mL^−1^, while that of 4APyCl was optimized to 1 mg mL^−1^ (Figs. S21 and S22).

To further validate the application potential of this synergistic surface engineering strategy, we fabricated devices with an FTO/Me-4PACz with 2-(9*H*-carbazol-9-yl)ethyl)phosphonic acid/3D/PDAI_2_/1D/PCBM/BCP/Ag structure. Effective light management and interfacial contact within the devices were achieved by utilizing FTO substrates with an anti-reflection coatings. The best-performing device achieved a champion PCE of 25.8% with a *V*_OC_ of 1.170 V, a *J*_SC_ of 26.0 mA cm^−2^, and an FF of 84.9% (Fig. 5f, Table S5), ranking it among the top-performing 3D/1D structured perovskite solar cells (Fig. 5g, Table S6). To study the effect of PDAI_2_/1D capping layer on the stability of PSCs, the unencapsulated devices are subjected to MPPT in accordance with the ISOS-L-1 protocol (Fig. 5h). Reduced impurity and the conformal 1D interfacial capping layer contribute to superior operational stability. Consequently, the device with a 3D/PDAI_2_/1D heterostructure retained 85% of its initial PCE after 1000 h of continuous illumination, compared to 64% for its 3D/1D counterpart and a mere 54% for the control device after just 500 h.

## Conclusions

In summary, highly ordered 1D perovskite capping layer was successfully fabricated on 3D/PDAI_2_. The pre-anchored PDAI_2_ not only provides field-effect passivation but also templates the subsequent vertical alignment of 1D Pb–I octahedral chains featuring continuous out-of-plane charge transport channels. Homogenized morphology and surface potential are realized through the conversion of excess PbI_2_ impurities into low-dimensional capping layer. The synergistic n-doping effect of PDAI_2_ and the 4APyCl optimizes the energy-level alignment between the modified 3D perovskite and the ETL, thereby collectively facilitating electron extraction and suppressing hole transfer. Consequently, the corresponding inverted PSCs deliver a champion power conversion efficiency of 25.8% and retain 85% of their initial efficiency after 1000 h MPPT in accordance with the ISOS-L-1 protocol. This leverages orientation engineering in the 1D perovskite to preserve the impurity healing benefits of the 1D capping layer while avoiding charge transport losses, thus advancing the prospects for fully realizing the potential of 3D/1D heterostructures.

## Supplementary Information

Below is the link to the electronic supplementary material.

Supplementary file1 (DOCX 33503 KB)

## References

[1] J. Du, J. Chen, B. Ouyang, A. Sun, C. Tian et al., Face-on oriented self-assembled molecules with enhanced π–π stacking for highly efficient inverted perovskite solar cells on rough FTO substrates. Energy Environ. Sci. **18**(7), 3196–3210 (2025). 10.1039/D4EE05849F

[2] X. Shen, X. Lin, H. Su, Z. Zhang, T. Wu et al., Key advancements and emerging trends of perovskite solar cells in 2024-2025. Nano-Micro Lett. **18**(1), 209 (2026). 10.1007/s40820-025-02022-6 10.1007/s40820-025-02022-6 PMC12804561 41535540

[3] Y. Liu, Z. Zhang, T. Wu, W. Xiang, Z. Qin et al., Cost effectivities analysis of perovskite solar cells: will it outperform crystalline silicon ones? Nano-Micro Lett. **17**(1), 219 (2025). 10.1007/s40820-025-01744-x 10.1007/s40820-025-01744-x PMC12000492 40232344

[4] B. Li, S. Li, J. Gong, X. Wu, Z. Li et al., Fundamental understanding of stability for halide perovskite photovoltaics: the importance of interfaces. Chem **10**(1), 35–47 (2024). 10.1016/j.chempr.2023.09.002

[5] H. Lee, T. Moon, Y. Lee, J. Kim, Structural mechanisms of quasi-2D perovskites for next-generation photovoltaics. Nano-Micro Lett. **17**(1), 139 (2025). 10.1007/s40820-024-01609-9 10.1007/s40820-024-01609-9 PMC11806192 39920413

[6] Y. Zhang, J. Xi, Y. Deng, W. Liu, Z. Li et al., The crucial role of organic ligands on 2D/3D perovskite solar cells: a comprehensive review. Adv. Energy Mater. **14**(48), 2403326 (2024). 10.1002/aenm.202403326

[7] Y. Chen, B. Liu, Q. Zhou, D. Ma, X. Han et al., Critical role of 1D materials in realizing efficient and stable perovskite solar cells. J. Mater. Chem. A **11**(35), 18592–18604 (2023). 10.1039/D3TA03174H

[8] H. Rafique, G. Abbas, M.J. Mendes, P. Barquinha, R. Martins et al., Recent advancements and perspectives of low-dimensional halide perovskites for visual perception and optoelectronic applications. Nano-Micro Lett. **18**(1), 44 (2025). 10.1007/s40820-025-01823-z 10.1007/s40820-025-01823-z PMC12381337 40856914

[9] X. Shen, X. Lin, Y. Peng, Y. Zhang, F. Long et al., Two-dimensional materials for highly efficient and stable perovskite solar cells. Nano-Micro Lett. **16**(1), 201 (2024). 10.1007/s40820-024-01417-1 10.1007/s40820-024-01417-1 PMC11116351 38782775

[10] K.T. Cho, G. Grancini, Y. Lee, E. Oveisi, J. Ryu et al., Selective growth of layered perovskites for stable and efficient photovoltaics. Energy Environ. Sci. **11**(4), 952–959 (2018). 10.1039/c7ee03513f

[11] P. Liu, X. Li, T. Cai, W. Xing, N. Yang et al., Molecular structure tailoring of organic spacers for high-performance Ruddlesden-Popper perovskite solar cells. Nano-Micro Lett. **17**(1), 35 (2024). 10.1007/s40820-024-01500-7 10.1007/s40820-024-01500-7 PMC11469732 39387997

[12] S. Sidhik, Y. Wang, M. De Siena, R. Asadpour, A.J. Torma et al., Deterministic fabrication of 3D/2D perovskite bilayer stacks for durable and efficient solar cells. Science **377**(6613), 1425–1430 (2022). 10.1126/science.abq7652 36137050 10.1126/science.abq7652

[13] X. Chang, R. Azmi, T. Yang, N. Wu, S.Y. Jeong et al., Solvent-dripping modulated 3D/2D heterostructures for high-performance perovskite solar cells. Nat. Commun. **16**(1), 1042 (2025). 10.1038/s41467-025-56409-5 39863604 10.1038/s41467-025-56409-5 PMC11763036

[14] M.-C. Shih, S. Tan, Y. Lu, T. Kodalle, D.-K. Lee et al., A 2D/3D heterostructure perovskite solar cell with a phase-pure and pristine 2D layer. Adv. Mater. **37**(17), 2416672 (2025). 10.1002/adma.202416672 40099622 10.1002/adma.202416672 PMC12038532

[15] P. Chen, D. He, X. Huang, C. Zhang, L. Wang, Bilayer 2D–3D perovskite heterostructures for efficient and stable solar cells. ACS Nano **18**(1), 67–88 (2024). 10.1021/acsnano.3c09176 38131195 10.1021/acsnano.3c09176

[16] L. Scalon, Y. Vaynzof, Multidimensional perovskite solar cells: what’s next after 3D/2D? Adv. Energy Mater. **15**(44), 2502686 (2025). 10.1002/aenm.202502686

[17] Y. Zhang, C. Li, E. Bi, T. Wang, P. Zhang et al., Efficient inverted perovskite solar cells with a low-dimensional halide/perovskite heterostructure. Adv. Energy Mater. **12**(48), 2202191 (2022). 10.1002/aenm.202202191

[18] D. Wang, J. Chen, P. Zhu, Y. Qiao, H. Hu et al., Binary microcrystal additives enabled antisolvent-free perovskite solar cells with high efficiency and stability. Adv. Energy Mater. **13**(7), 2203649 (2023). 10.1002/aenm.202203649

[19] J. Wang, L. Liu, S. Chen, L. Qi, M. Zhao et al., Growth of 1D nanorod perovskite for surface passivation in FAPbI3 perovskite solar cells. Small **18**(3), 2104100 (2022). 10.1002/smll.202104100 10.1002/smll.202104100 34738722

[20] X. Chang, Y. Liu, Y. Ping, N. Wu, T. Yang et al., Multivalent ligands regulate dimensional engineering for inverted perovskite solar modules. Science **391**(6781), 153–159 (2026). 10.1126/science.aea0656 41505535 10.1126/science.aea0656

[21] Y. Zou, Y. Zhao, Reconfiguring perovskite interlayers. Nat. Mater. **25**(2), 166–167 (2026). 10.1038/s41563-025-02438-w 41402483 10.1038/s41563-025-02438-w

[22] T. Kong, H. Xie, Y. Zhang, J. Song, Y. Li et al., Perovskitoid-templated formation of a 1D@3D perovskite structure toward highly efficient and stable perovskite solar cells. Adv. Energy Mater. **11**(34), 2101018 (2021). 10.1002/aenm.202101018

[23] S. Li, H. Gu, A. Zhu, J. Guo, C. Xi et al., Anion-cation synergistic regulation of low-dimensional perovskite passivation layer for perovskite solar cells. Adv. Mater. **37**(28), 2500988 (2025). 10.1002/adma.202500988 40270282 10.1002/adma.202500988 PMC12272002

[24] F. Wang, D. Duan, K. Zhou, Y.Z.B. Xue, X. Liang et al., Ionic liquid engineering enabled in-plane orientated 1D perovskite nanorods for efficient mixed-dimensional perovskite photovoltaics. InfoMat **5**(8), e12459 (2023). 10.1002/inf2.12459

[25] F. Ye, T. Tian, J. Su, R. Jiang, J. Li et al., Tailoring low-dimensional perovskites passivation for efficient two-step-processed FAPbI3 solar cells and modules. Adv. Energy Mater. **14**(4), 2302775 (2024). 10.1002/aenm.202302775

[26] X. Zhou, X. Liang, F. Wang, H. Sun, Q. Zhu et al., Pyridine substitution strategy for one-dimensional perovskite: toward efficient and stable mixed-dimensional photovoltaics. Chem. Eng. J. **493**, 152539 (2024). 10.1016/j.cej.2024.152539

[27] Y. Yang, H. Chen, C. Liu, J. Xu, C. Huang et al., Amidination of ligands for chemical and field-effect passivation stabilizes perovskite solar cells. Science **386**(6724), 898–902 (2024). 10.1126/science.adr2091 39571031 10.1126/science.adr2091

[28] P. Shi, B. Ding, D. Jin, M. Oner, X. Zhang et al., Micro-homogeneity of lateral energy landscapes governs the performance in perovskite solar cells. Nat. Commun. **15**(1), 9703 (2024). 10.1038/s41467-024-53953-4 39516477 10.1038/s41467-024-53953-4 PMC11549436

[29] X. Zheng, S. Ahmed, Y. Zhang, G. Xu, J. Wang et al., Differentiating the 2D passivation from amorphous passivation in perovskite solar cells. Nano-Micro Lett. **18**(1), 62 (2025). 10.1007/s40820-025-01913-y 10.1007/s40820-025-01913-y PMC12420534 40924235

[30] S. You, F.T. Eickemeyer, J. Gao, J.-H. Yum, X. Zheng et al., Bifunctional hole-shuttle molecule for improved interfacial energy level alignment and defect passivation in perovskite solar cells. Nat. Energy **8**(5), 515–525 (2023). 10.1038/s41560-023-01249-0

[31] R.T. Ross, Some thermodynamics of photochemical systems. J. Chem. Phys. **46**(12), 4590–4593 (1967). 10.1063/1.1840606

[32] C. Liu, Y. Yang, H. Chen, J. Xu, A. Liu et al., Bimolecularly passivated interface enables efficient and stable inverted perovskite solar cells. Science **382**(6672), 810–815 (2023). 10.1126/science.adk1633 37972154 10.1126/science.adk1633

[33] H. Chen, A. Maxwell, C. Li, S. Teale, B. Chen et al., Regulating surface potential maximizes voltage in all-perovskite tandems. Nature **613**(7945), 676–681 (2023). 10.1038/s41586-022-05541-z 36379225 10.1038/s41586-022-05541-z

[34] Y. Guo, F. Yao, Y. Zhang, G. Chen, S. Du et al., A universal surface fixed charge reconstruction strategy to minimize contact loss in wide bandgap perovskite photovoltaics. Energy Environ. Sci. **18**(10), 4916–4924 (2025). 10.1039/D4EE05855K

[35] J. Wang, L. Zeng, D. Zhang, A. Maxwell, H. Chen et al., Halide homogenization for low energy loss in 2-eV-bandgap perovskites and increased efficiency in all-perovskite triple-junction solar cells. Nat. Energy **9**(1), 70–80 (2024). 10.1038/s41560-023-01406-5

[36] Y. Zou, J. Liu, Y. Chang, C. Duan, W. Han et al., Differential ligand–cation interactions enable 2D-template-induced ordered assembly for efficient tin-based perovskite photovoltaics. Adv. Mater. **38**(12), e22717 (2026). 10.1002/adma.202522717 41543203 10.1002/adma.202522717

[37] A. Diercks, J. Petry, T. Feeney, R. Singh, T. Zhao et al., Sequential evaporation of inverted FAPbI_3_ perovskite solar cells–impact of substrate on crystallization and film formation. ACS Energy Lett. **10**(3), 1165–1173 (2025). 10.1021/acsenergylett.4c03315

[38] B. Jiao, L. Tan, Y. Ye, N. Ren, M. Li et al., One-stone-two-birds: over 26% efficiency in perovskite solar cells *via* synergistic crystallization & interface regulation. Energy Environ. Sci. **18**(11), 5437–5447 (2025). 10.1039/D5EE00189G

[39] H. Wang, S. Su, Y. Chen, M. Ren, S. Wang et al., Impurity-healing interface engineering for efficient perovskite submodules. Nature **634**(8036), 1091–1095 (2024). 10.1038/s41586-024-08073-w 39326517 10.1038/s41586-024-08073-w

[40] Y. Zhao, F. Ma, Z. Qu, S. Yu, T. Shen et al., Inactive (PbI_2_)_2_RbCl stabilizes perovskite films for efficient solar cells. Science **377**(6605), 531–534 (2022). 10.1126/science.abp8873 35901131 10.1126/science.abp8873

[41] Y. Zou, W. Yu, H. Guo, Q. Li, X. Li et al., A crystal capping layer for formation of black-phase FAPbI_3_ perovskite in humid air. Science **385**(6705), 161–167 (2024). 10.1126/science.adn9646 38991067 10.1126/science.adn9646

[42] Y. Wen, T. Zhang, X. Wang, T. Liu, Y. Wang et al., Amorphous (lysine)_2_PbI_2_ layer enhanced perovskite photovoltaics. Nat. Commun. **15**(1), 7085 (2024). 10.1038/s41467-024-51551-y 39154032 10.1038/s41467-024-51551-y PMC11330473

[43] J. Wu, Y. Li, Y. Zhang, Y. Li, Y. Huang et al., Highly orientational order perovskite induced by in situ-generated 1D perovskitoid for efficient and stable printable photovoltaics. Small **18**(19), 2200130 (2022). 10.1002/smll.202200130 10.1002/smll.202200130 35403377

[44] M. Lu, J. Ding, Q. Ma, Z. Zhang, M. Li et al., Dual-site passivation by heterocycle functionalized amidinium cations toward high-performance inverted perovskite solar cells and modules. Energy Environ. Sci. **18**(12), 5973–5984 (2025). 10.1039/d5ee00524h

[45] L. Yang, Z. Fang, Y. Jin, H. Feng, B. Deng et al., Suppressing halide segregation *via* Pyridine-derivative isomers enables efficient 1.68 eV bandgap perovskite solar cells. Adv. Mater. **36**(21), 2311923 (2024). 10.1002/adma.202311923 10.1002/adma.202311923 38400811

[46] Y. Xu, J. Yu, S. Liu, F. Tang, N. Ma et al., Surface potential homogenization improves perovskite solar cell performance. Adv. Energy Mater. **15**(12), 2404755 (2025). 10.1002/aenm.202404755

[47] M. Li, J. Ding, Z. Zhang, Q. Ma, C. Li et al., Functional group engineering stabilizing precursor solution and passivating defects for operationally stable and highly reproducible inverted perovskite solar cells. Adv. Mater. **37**(27), 2502729 (2025). 10.1002/adma.202502729 10.1002/adma.202502729 40331475

[48] H. Zheng, G. Liu, X. Dong, F. Chen, C. Wang et al., Self-regulated bilateral anchoring enables efficient charge transport pathways for high-performance rigid and flexible perovskite solar cells. Nano-Micro Lett. **17**(1), 328 (2025). 10.1007/s40820-025-01846-6 10.1007/s40820-025-01846-6 PMC12259523 40658297

[49] Y.-H. Huang, S.-Y. Zou, C.-Y. Sheng, Y.-C. Fang, X.-D. Wang et al., Lattice anchoring stabilizes α-FAPbI_3_ perovskite for high-performance X-ray detectors. Nano-Micro Lett. **18**(1), 14 (2025). 10.1007/s40820-025-01856-4 10.1007/s40820-025-01856-4 PMC12307842 40728710

[50] H. Chen, S. Teale, B. Chen, Y. Hou, L. Grater et al., Quantum-size-tuned heterostructures enable efficient and stable inverted perovskite solar cells. Nat. Photon. **16**(5), 352–358 (2022). 10.1038/s41566-022-00985-1

[51] Y. Liu, T. Ma, C. Wang, Z. Yang, Y. Zhao et al., Synergistic immobilization of ions in mixed tin-lead and all-perovskite tandem solar cells. Nat. Commun. **16**(1), 3477 (2025). 10.1038/s41467-025-58810-6 40216812 10.1038/s41467-025-58810-6 PMC11992112

[52] M. Stolterfoht, C.M. Wolff, J.A. Márquez, S. Zhang, C.J. Hages et al., Visualization and suppression of interfacial recombination for high-efficiency large-area pin perovskite solar cells. Nat. Energy **3**(10), 847–854 (2018). 10.1038/s41560-018-0219-8

[53] S.D. Stranks, R.L.Z. Hoye, D. Di, R.H. Friend, F. Deschler, The physics of light emission in halide perovskite devices. Adv. Mater. **31**(47), 1803336 (2019). 10.1002/adma.201803336 10.1002/adma.201803336 30187974

[54] J. Warby, F. Zu, S. Zeiske, E. Gutierrez-Partida, L. Frohloff et al., Understanding performance limiting interfacial recombination in pin perovskite solar cells. Adv. Energy Mater. **12**(12), 2103567 (2022). 10.1002/aenm.202103567

[55] C. Geng, K. Zhang, C. Wang, C.H. Wu, J. Jiang et al., Crystallization modulation and holistic passivation enables efficient two-terminal perovskite/CuIn(Ga)Se_2_ tandem solar cells. Nano-Micro Lett. **17**(1), 8 (2024). 10.1007/s40820-024-01514-1 10.1007/s40820-024-01514-1 PMC11416436 39306599

[56] X. Zang, S. Xiong, S. Jiang, D. Li, H. Wu et al., Passivating dipole layer bridged 3D/2D perovskite heterojunction for highly efficient and stable p-i-n solar cells. Adv. Mater. **36**(13), 2309991 (2024). 10.1002/adma.202309991 10.1002/adma.202309991 38154115

[57] X. Zhang, F. Liu, Y. Guan, Y. Zou, C. Wu et al., Reducing the Voc loss of hole transport layer-free carbon-based perovskite solar cells *via* dual interfacial passivation. Nano-Micro Lett. **17**(1), 258 (2025). 10.1007/s40820-025-01775-4 10.1007/s40820-025-01775-4 PMC12089553 40387983

