# Supplementary material for: Diamine-Mediated Synergistic Engineering of Orientation and Interfacial Field of 3D/1D Heterojunctions for Efficient Perovskite Photovoltaics
Source: Nanomicro Lett. 2026 Apr 14;18:330. doi: 10.1007/s40820-026-02190-z (PMC13079243; doi:10.1007/s40820-026-02190-z)
Supplement: Supplementary file 1 — Supplementary file1 (DOCX 33503 KB) [file 40820_2026_2190_MOESM1_ESM.docx]

Supporting Information for

**Diamine-Mediated Synergistic Engineering of Orientation and Interfacial Field of 3D/1D Heterojunctions for Efficient Perovskite Photovoltaics**

Yaobin Li^1#^, Yunxuan Cao^1#^, Yu Zou^1,2^*, Wenjin Yu^4^, Zhenhuang Su^5^, Zhuoer Cai^6^, Yueli Liu^1^, Qinyun Liu^1^, Hantao Wang^1^, Lefan Gong^1^, Yucheng Ye^1^, Rong Tang^1^, Yunan Gao^1^, Felix Thomas Eickemeyer^4^, Bo Qu^1^*, Lixin Xiao^1,3^*, Zhijian Chen^1^*

^1^State Key Laboratory for Artificial Microstructures and Mesoscopic Physics, School of Physics, Peking University, Beijing 100871, P. R. China

^2^Future Photovoltaics Research Center, Global Institute of Future Technology (GIFT), Shanghai Jiao Tong University, Shanghai 200240, P. R. China

^3^AI for Science (AI4S)-Preferred Program, Peking University Shenzhen Graduate School, Shenzhen 518055, P. R. China

^4^Laboratory of Photonics and Interfaces, Institute of Chemical Sciences and Engineering, École Polytechnique Fédérale de Lausanne (EPFL), Lausanne, Switzerland

^5^Shanghai Synchrotron Radiation Facility (SSRF), Shanghai Advanced Research Institute, Chinese Academy of Sciences, Shanghai 201204, P. R. China

^6^School of Chemistry and Chemical Engineering, Southeast University, Nanjing 211189, P. R. China

^#^Yaobin Li and Yunxuan Cao contributed equally to this work.

*Corresponding authors. E-mail: [yu.zou@sjtu.edu.cn](mailto:yu.zou@sjtu.edu.cn) (Yu Zou); [bqu@pku.edu.cn](mailto:bqu@pku.edu.cn) (Bo Qu); [lxxiao@pku.edu.cn](mailto:lxxiao@pku.edu.cn) (Lixin Xiao); [zjchen@pku.edu.cn](mailto:zjchen@pku.edu.cn) (Zhijian Chen)

**Supplementary Figures and Tables**


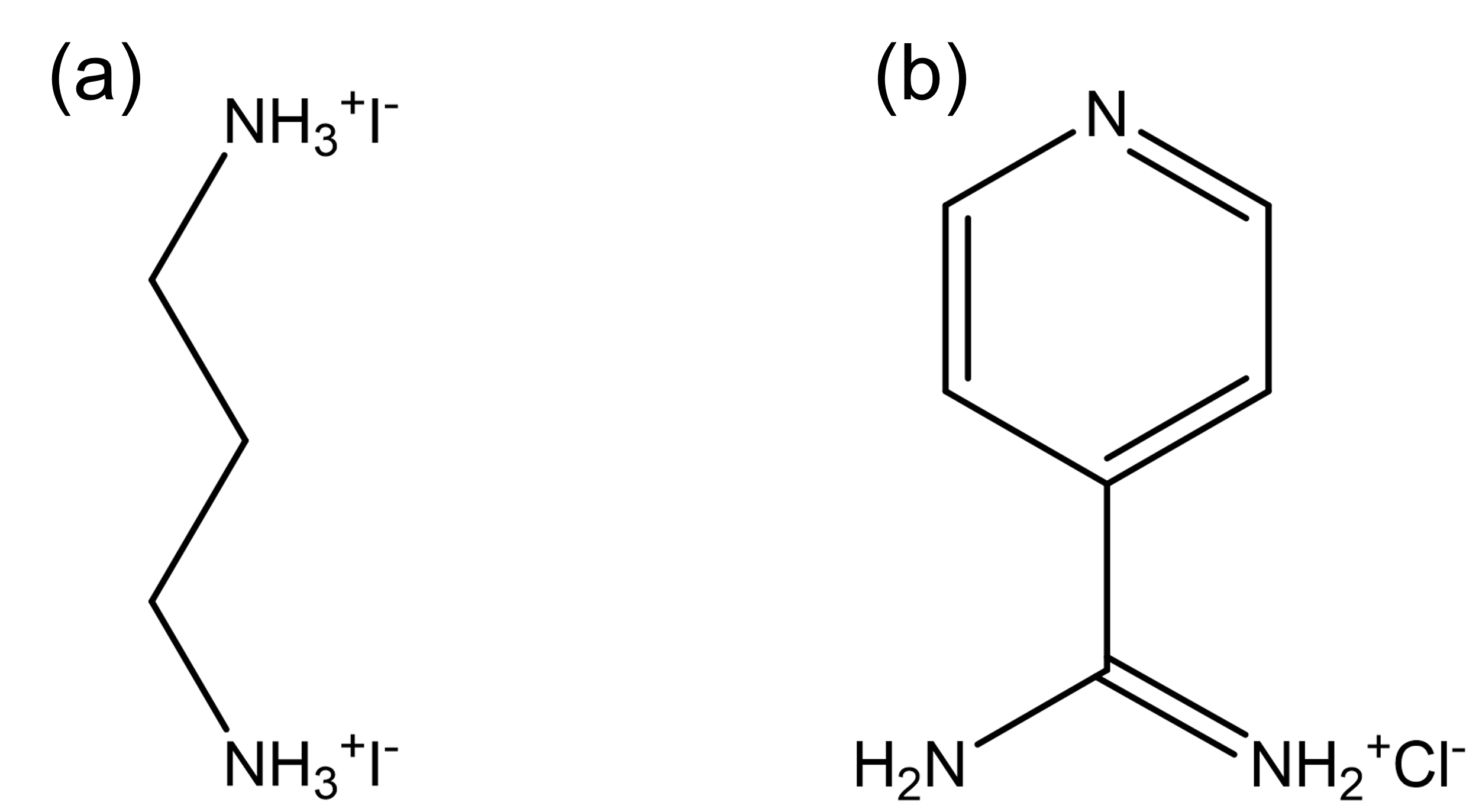


**Fig. S1** Chemical Structures of PDAI_2_ (a) and 4APyCl (b)


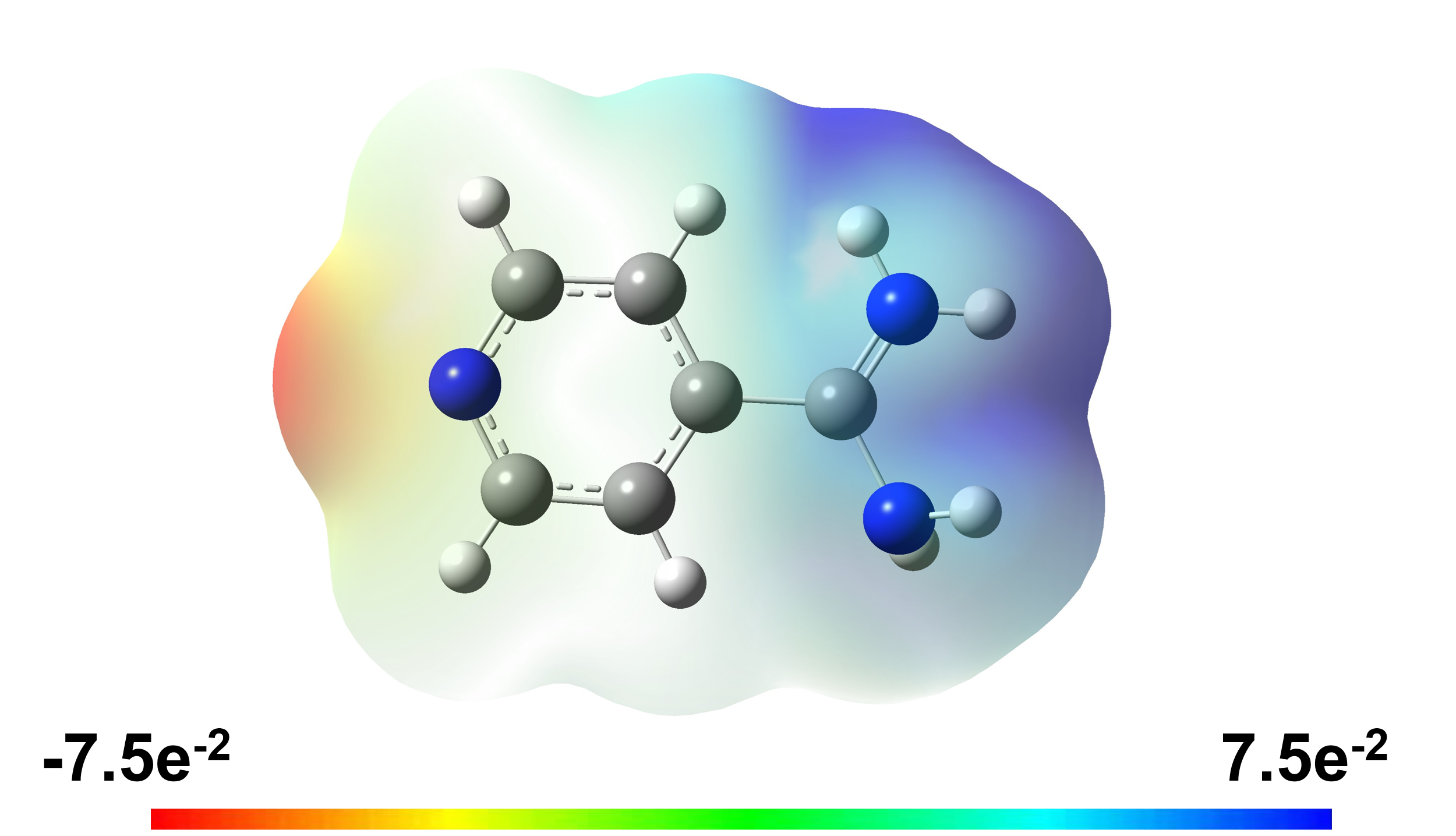


**Fig. S2** Gaussian electrostatic potential of 4APy^+^


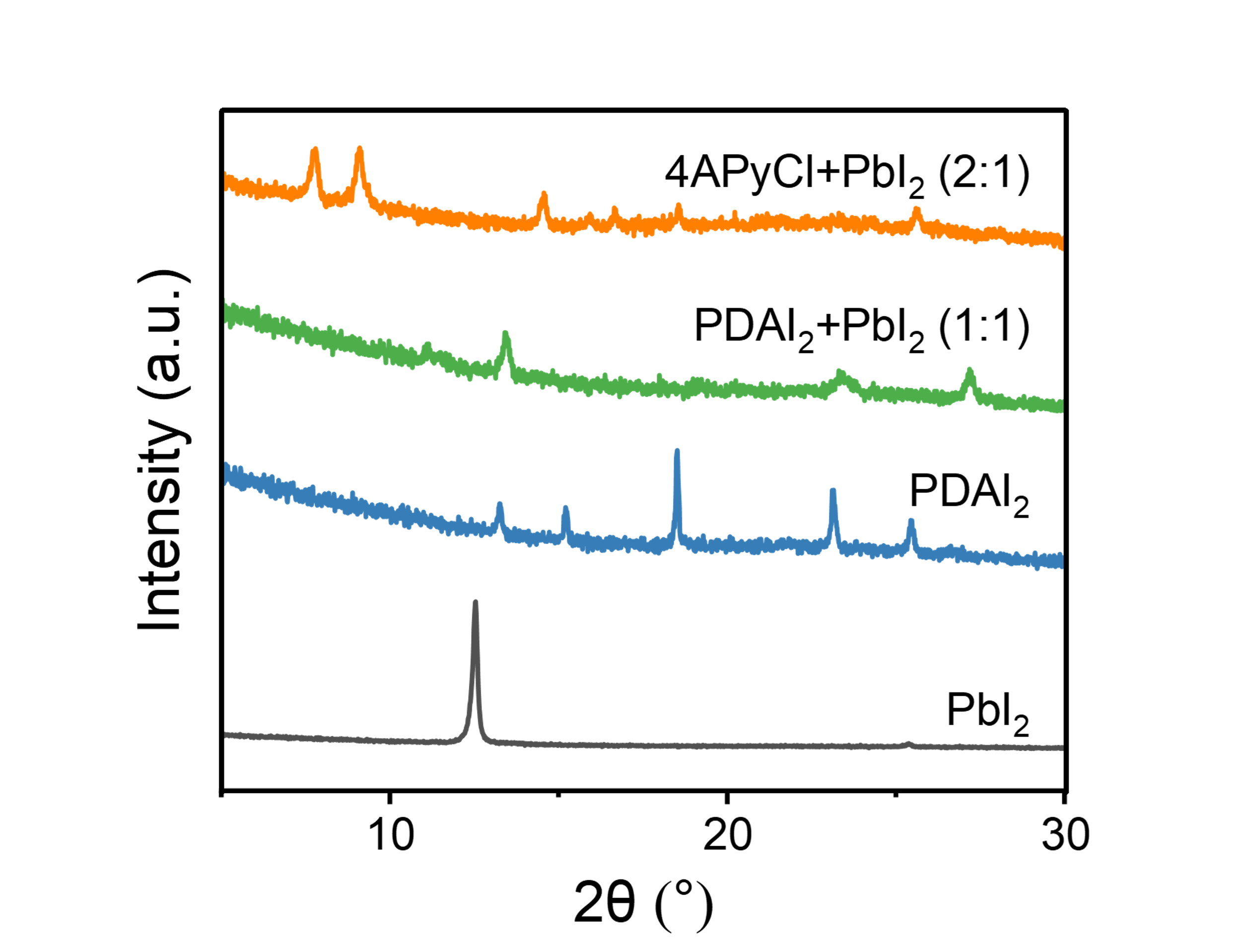


**Fig. S3** XRD of PbI_2_, PDAI_2_, PDAI_2_:PbI_2_ mixture and 4APyCl:PbI_2_ mixture


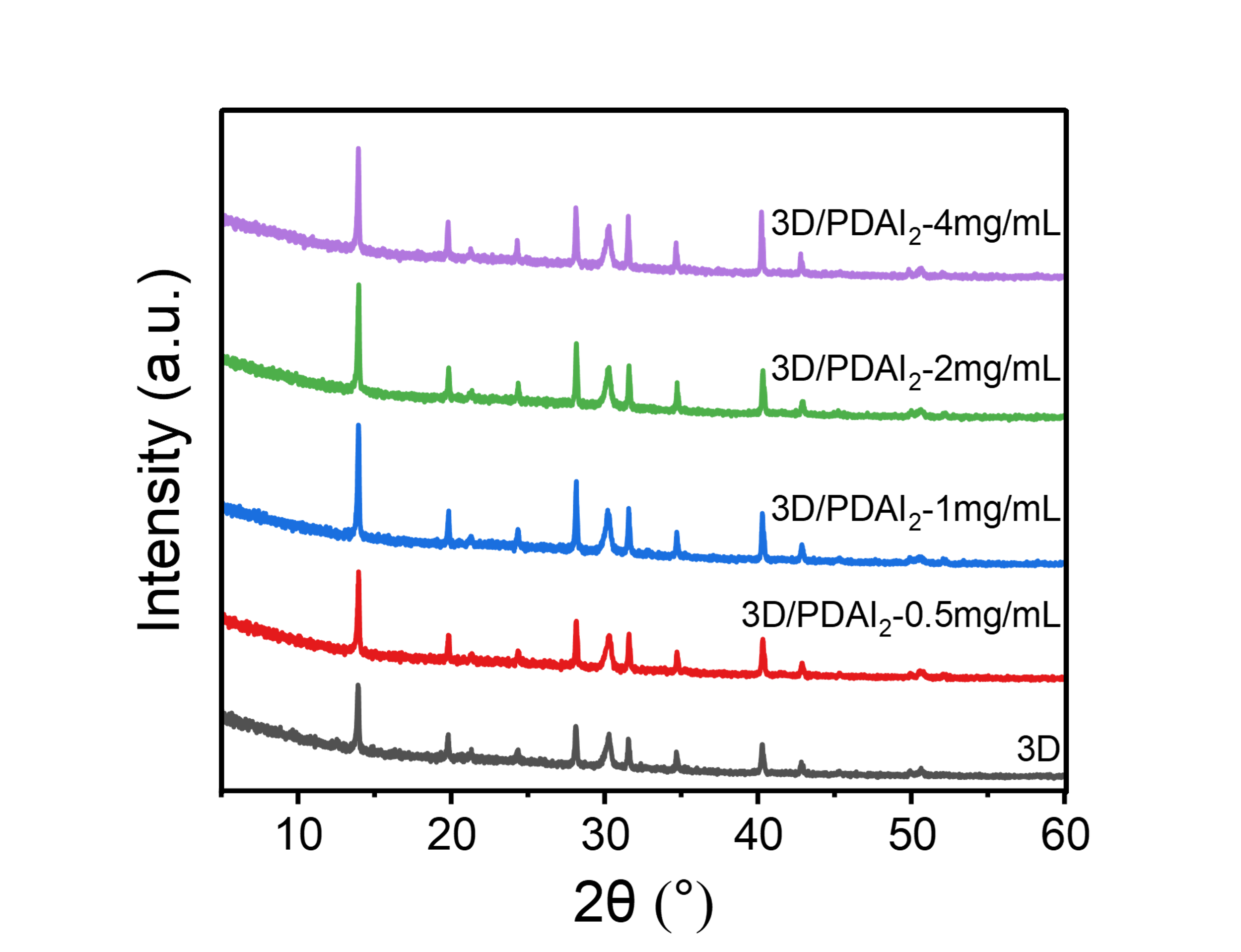


**Fig. S4** XRD patterns of 3D/PDAI_2_ with different concentration of PDAI_2_

**Fig. S5** XRD of PbI_2_, 4APyCl, 4APyCl:PbI_2_ mixture


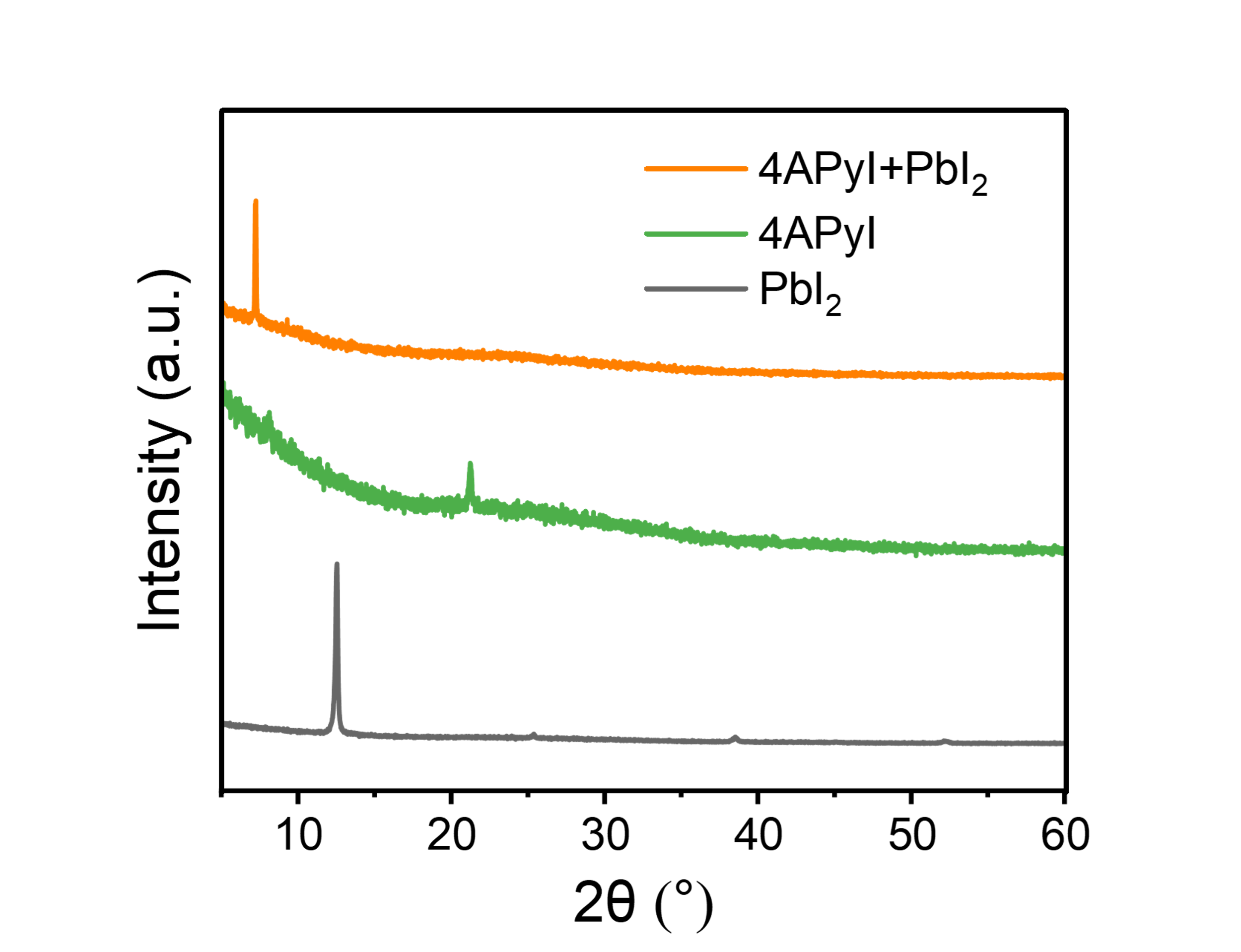


**Fig. S6** XRD of PbI_2_, 4APyI, 4APyI:PbI_2_ mixture


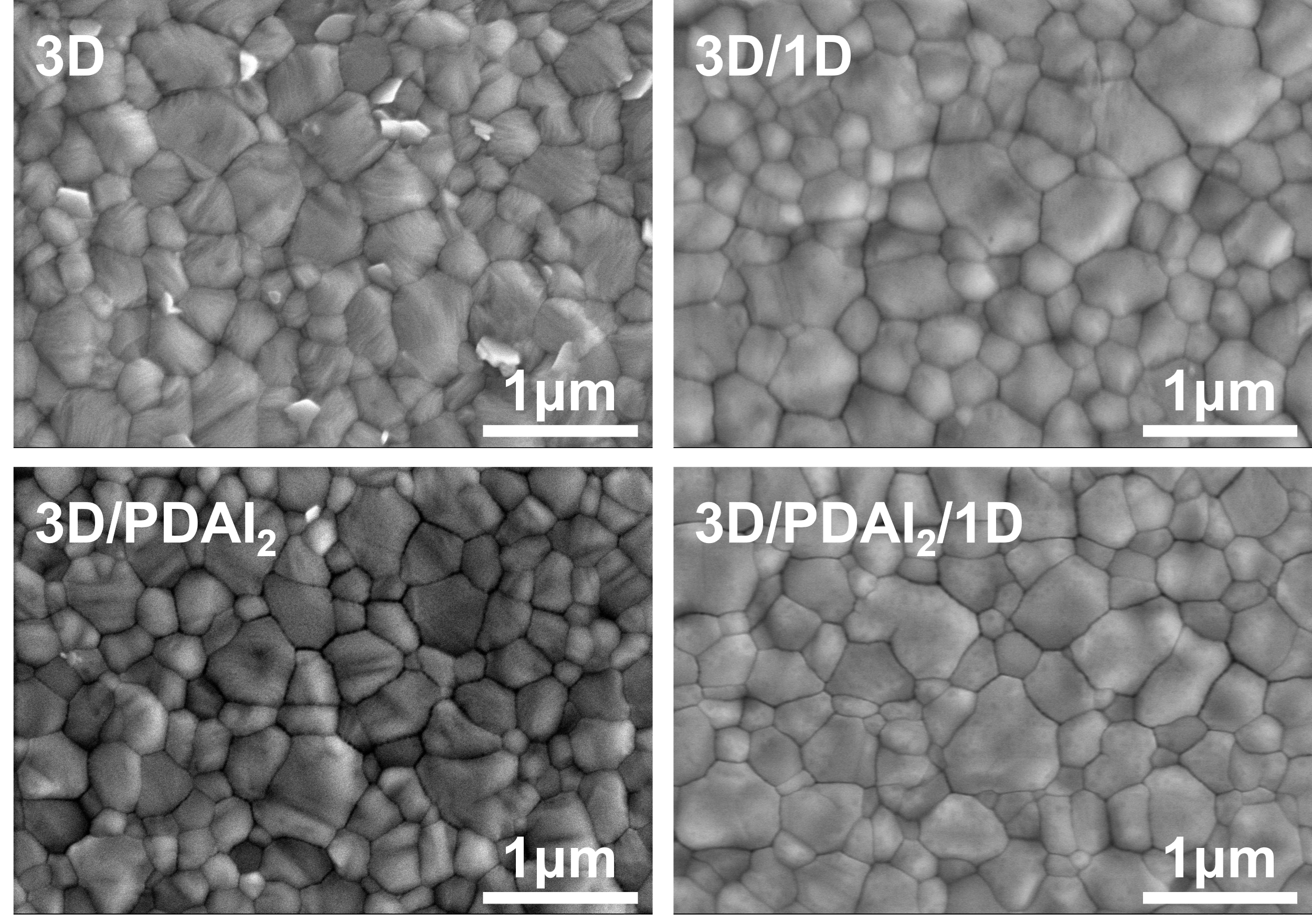


**Fig. S7** SEM images of perovskites with different post-treatments


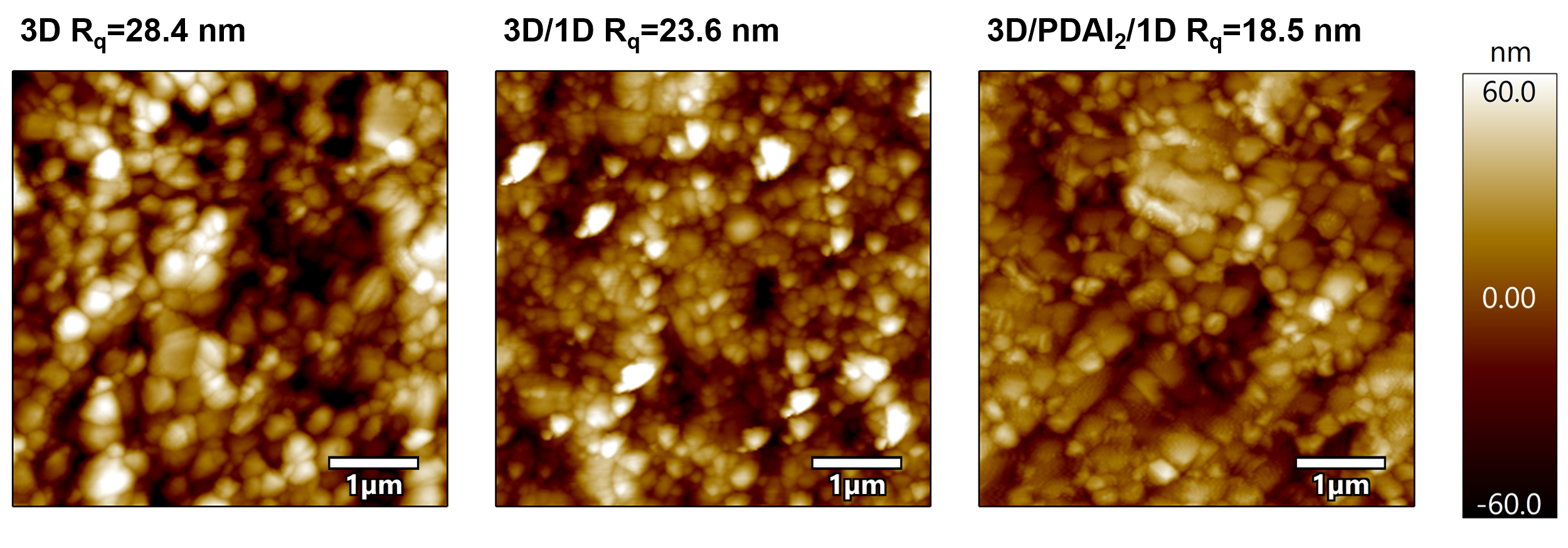


**Fig. S8** AFM images of perovskite films with different post-treatments


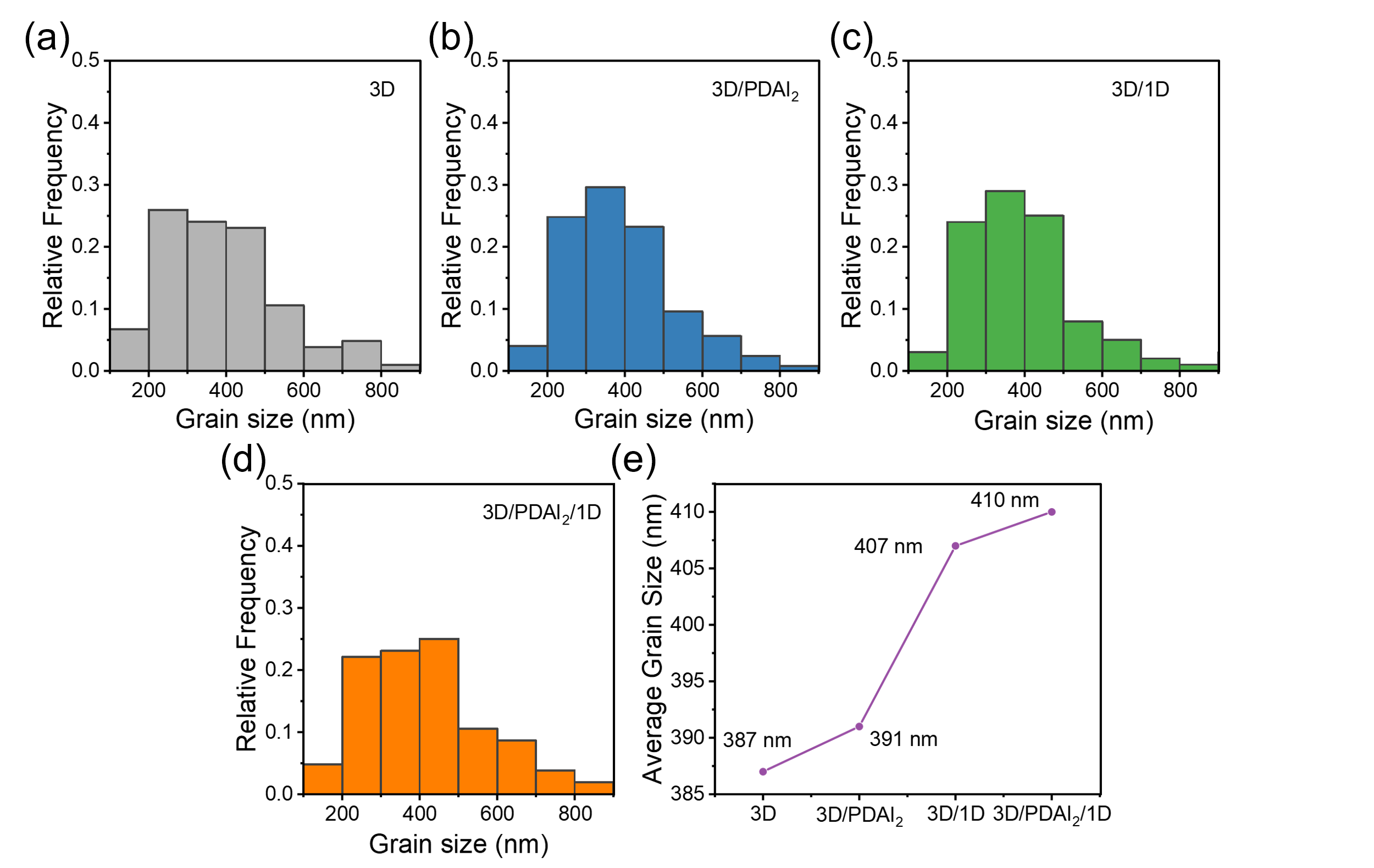


**Fig. S9** Grain size distribution and average grain size of perovskite films with different post-treatments


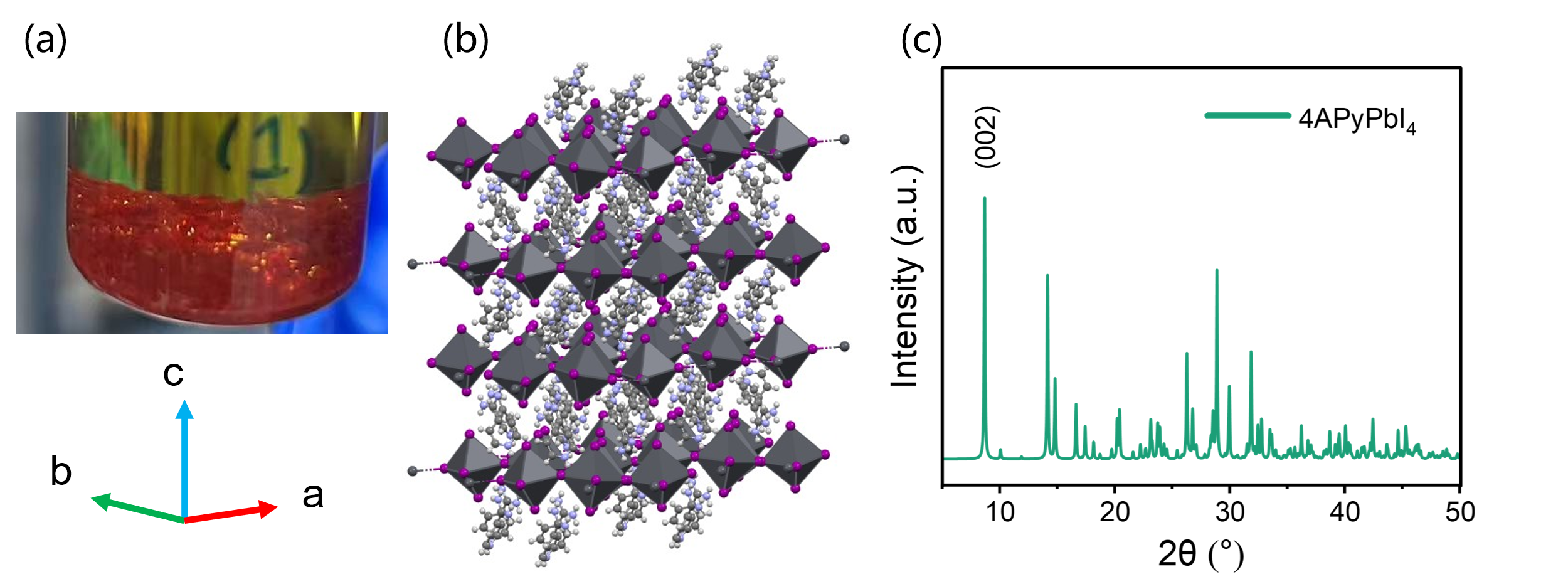


**Fig. S10** Crystal structure of 2D perovskite 4APyPbI_4_

**Note S1** During the crystal synthesis, HI further protonates the nitrogen atom on the pyridine ring of 4APy^+^, converting it into 4APy^2+^, which then reacts with PbI_2_ to form the DJ-type 2D perovskite 4APyPbI_4_. In contrast, DMF does not protonate 4APy^+^ during the synthesis process, resulting in the formation of slender 1D perovskite crystals, (4APy)_2_PbI_4_. The single-crystal XRD results of (4APy)_2_PbI_4_ are consistent with the GIWAXS data of the 3D/4APyCl sample (Fig. 2e).


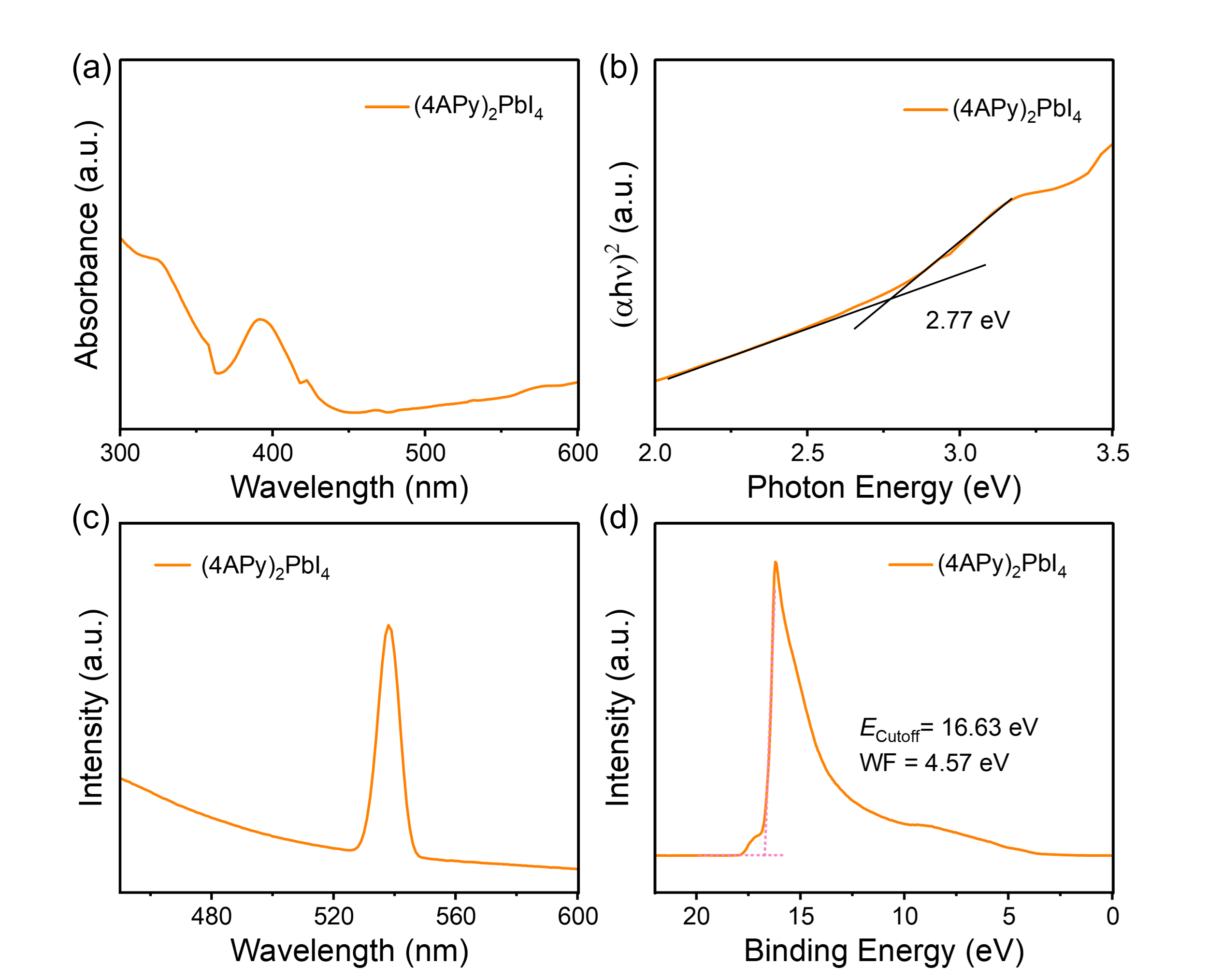


**Fig. S11** The photoelectric properties of the 1D perovskite (4APy)_2_PbI_4_


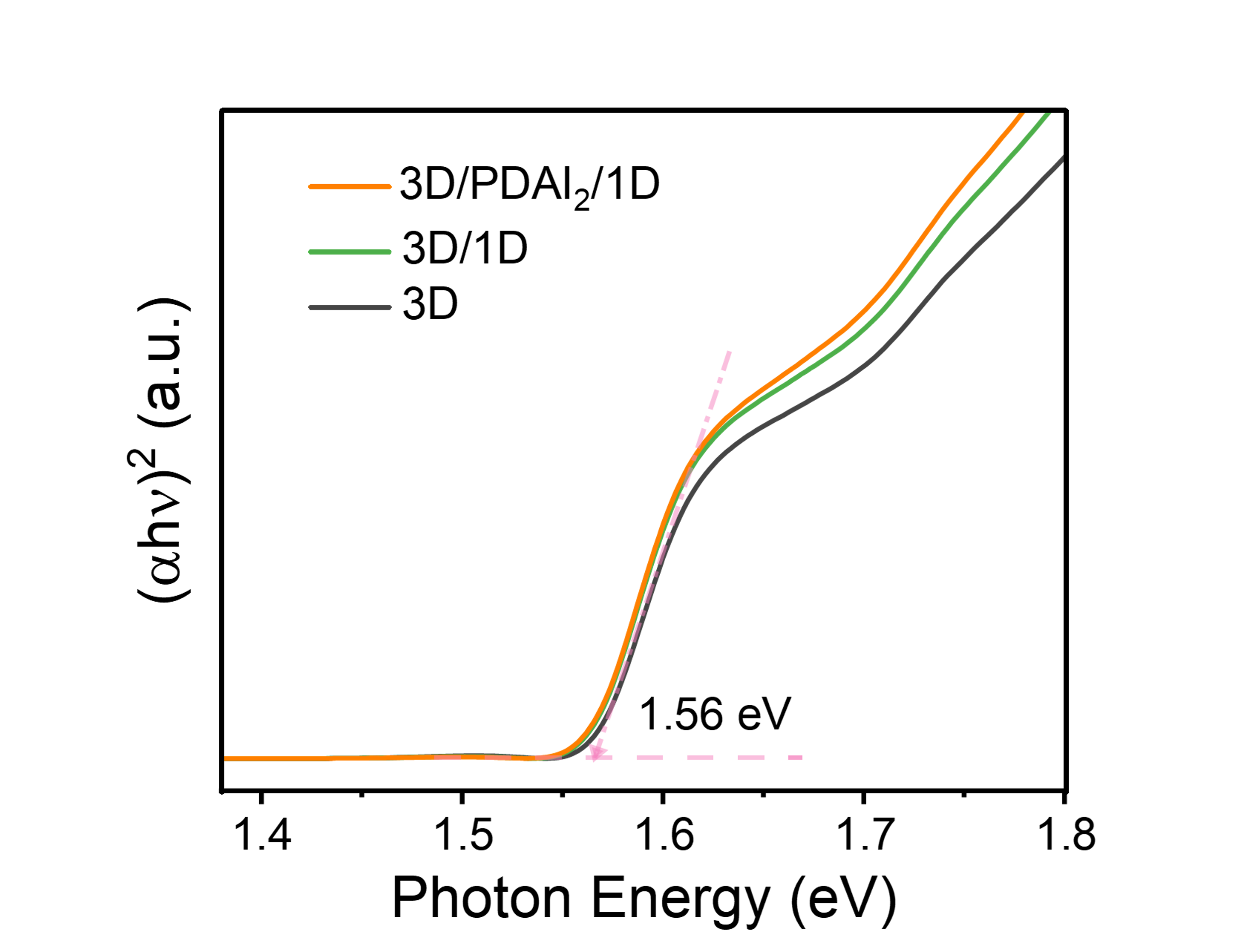


**Fig. S12** Tauc plot of perovskites with different post-treatment


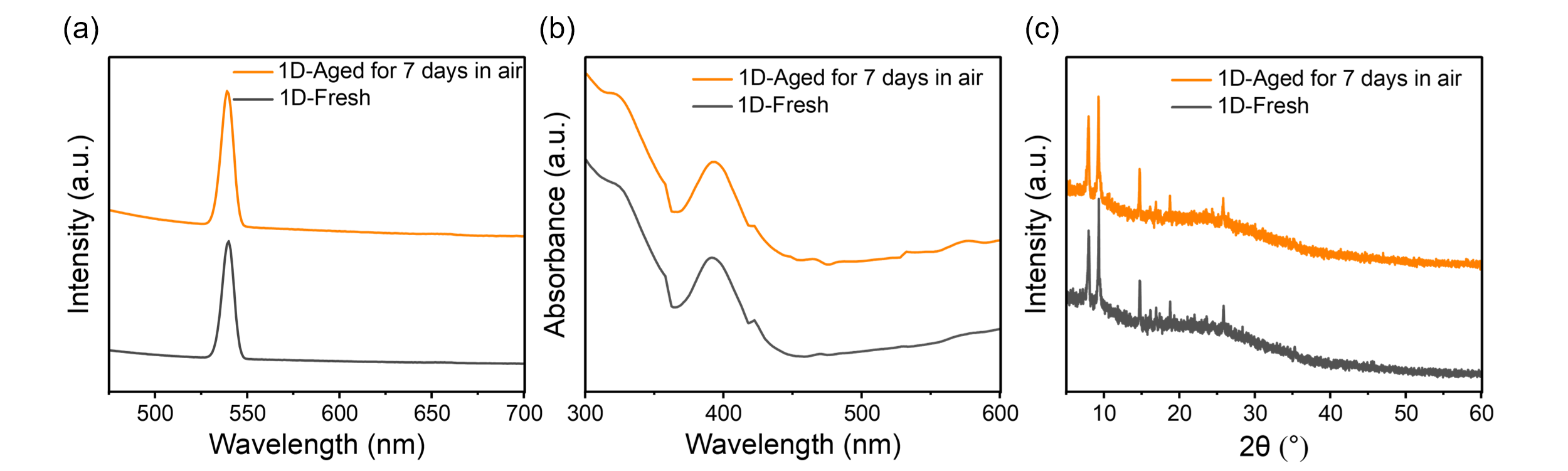


**Fig. S13** The stability of 1D perovskite (4APy)_2_PbI_4_ in air


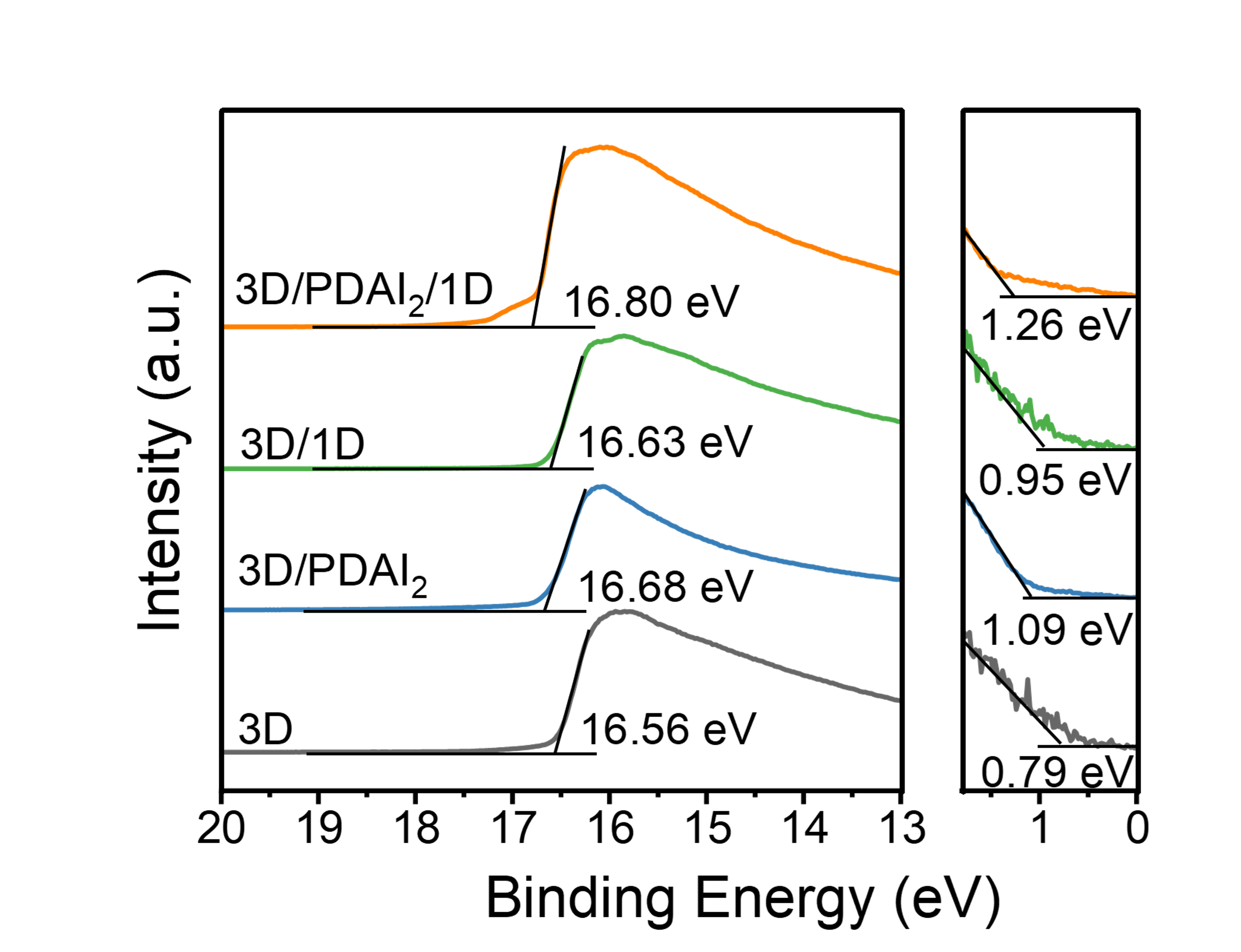


**Fig. S14** UPS results of perovskite films with different post-treatments


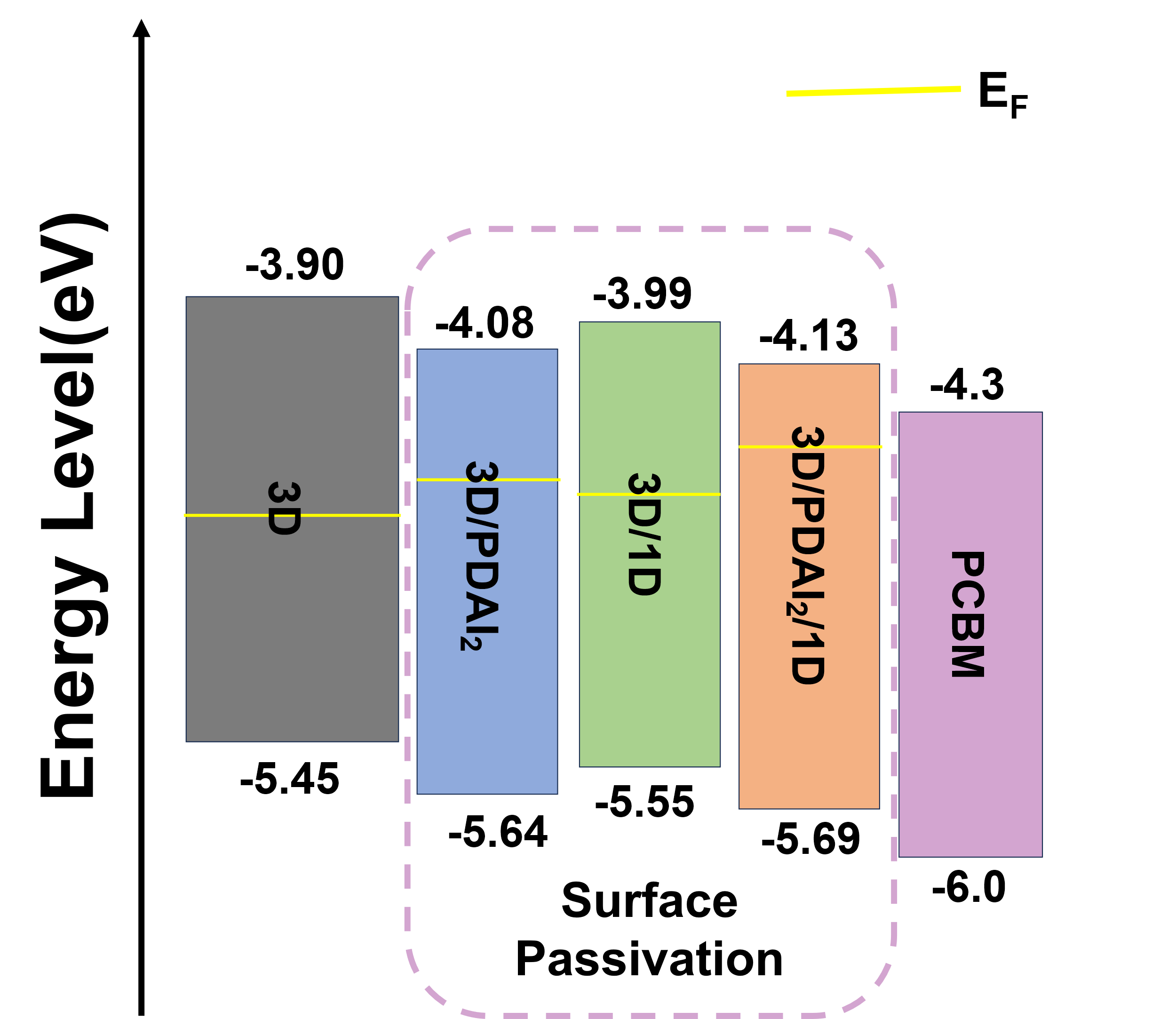


**Fig. S15** Energy band structure diagram with a unified vacuum energy level


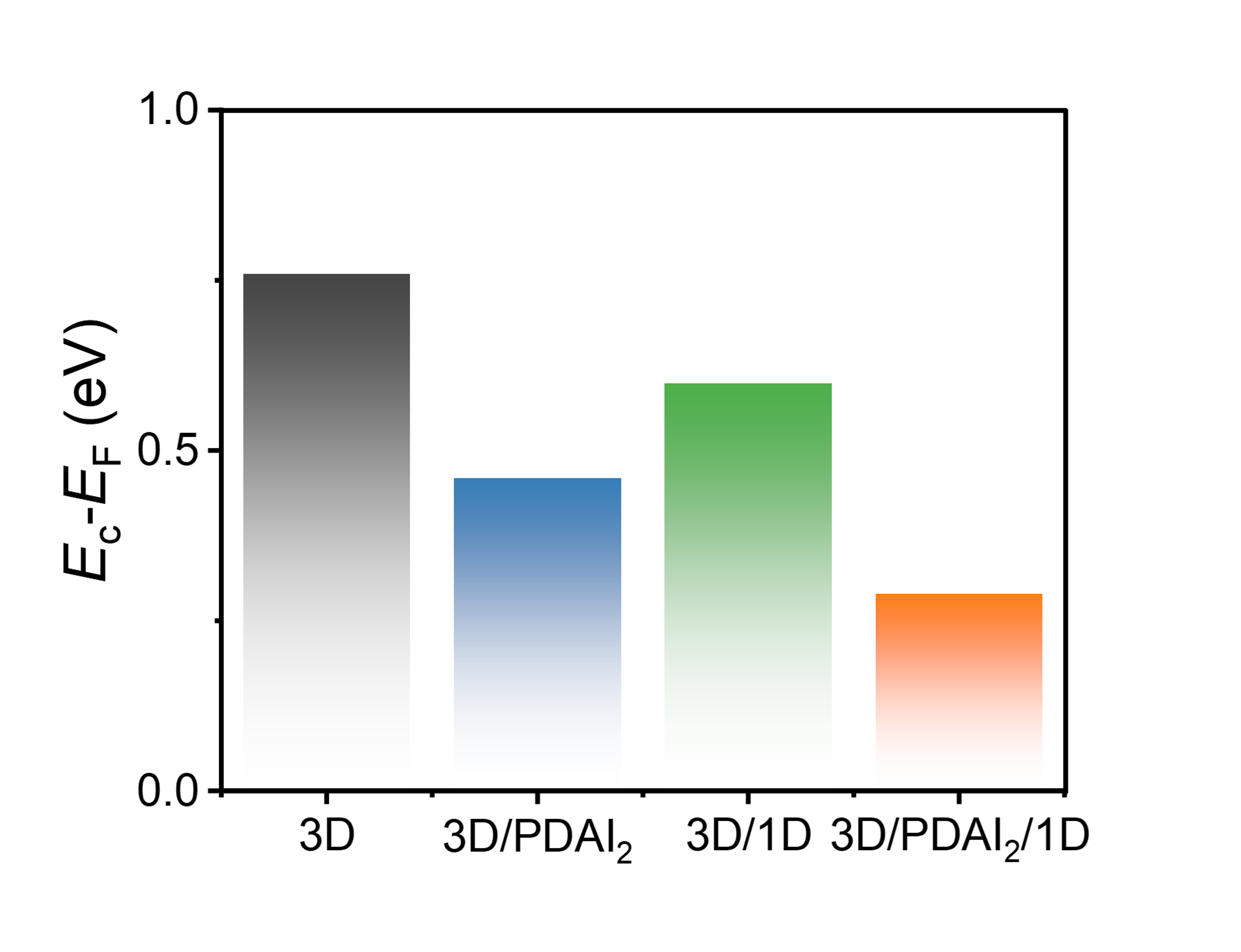


**Fig. S16** *E*_C_-*E*_F_ value of perovskite films with different post-treatments


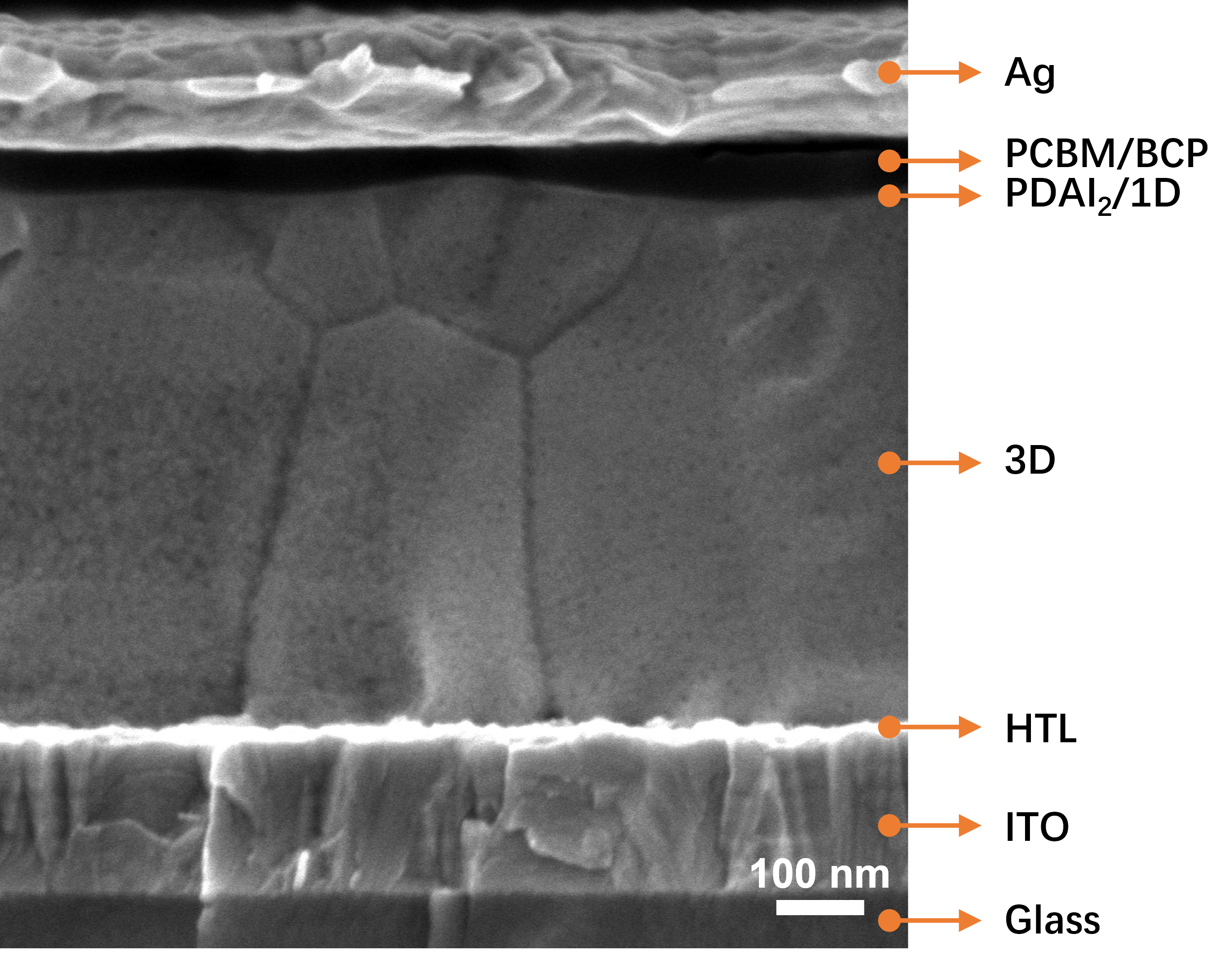


**Fig. S17** Cross-sectional SEM image of device of Figure 5a


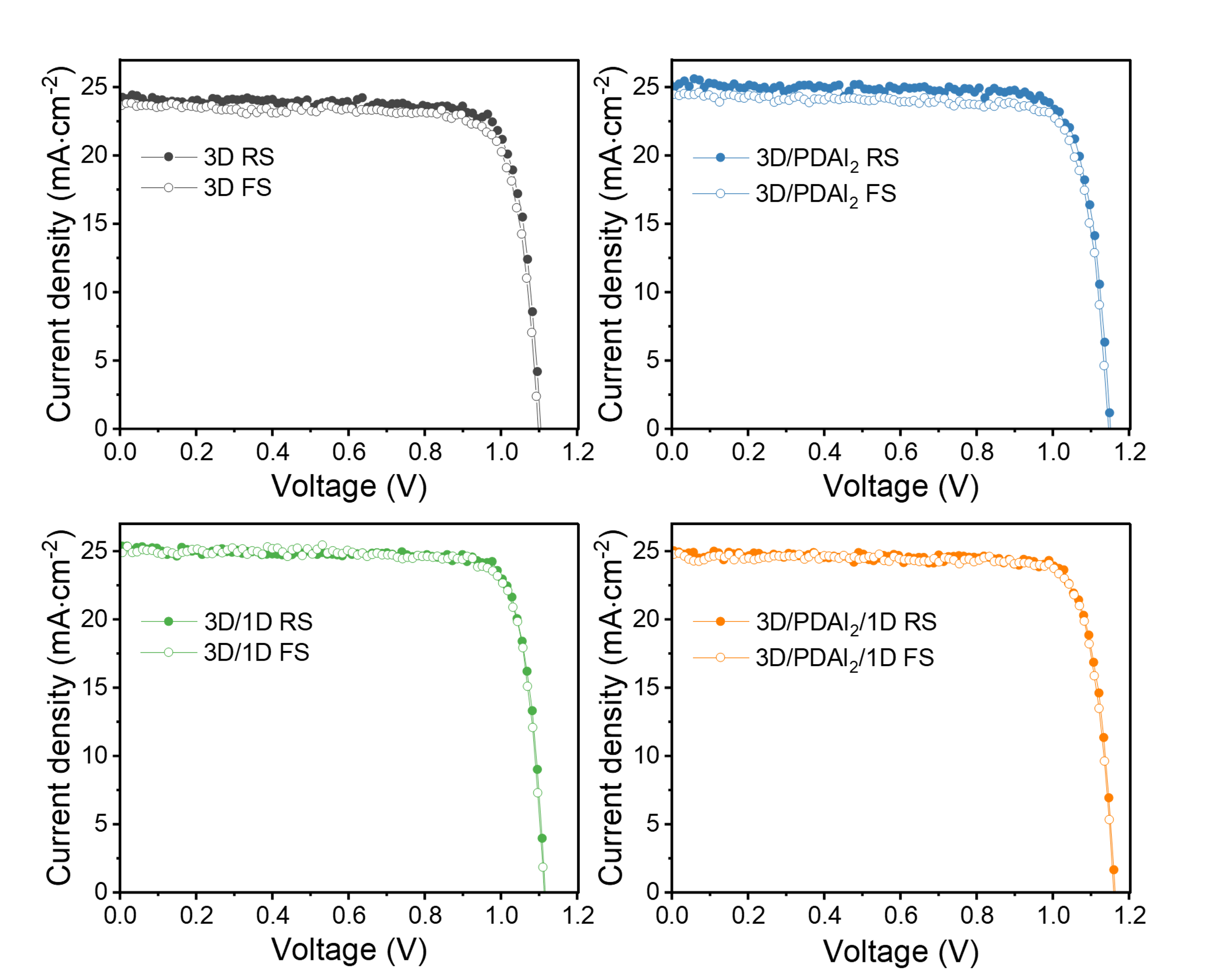


**Fig. S18** Reverse and forward *J*-*V* scan of champion PSCs with 4 structures of Figure 5b


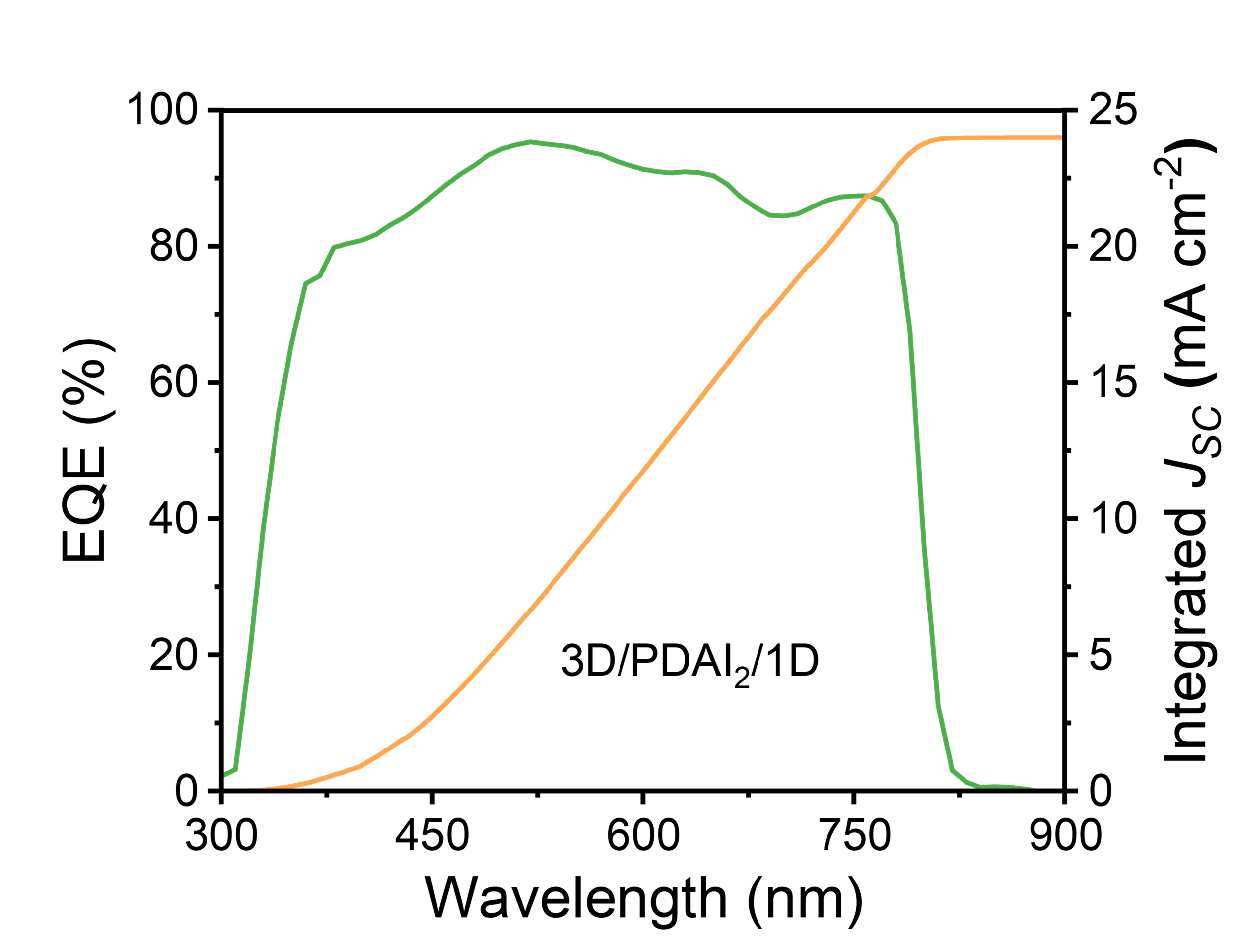


**Fig. S19** EQE curve of PSC with 3D/PDAI_2_/1D heterojunction of Figure 5b (Integral *J*_SC_ is 24.00 mA cm^-2^)


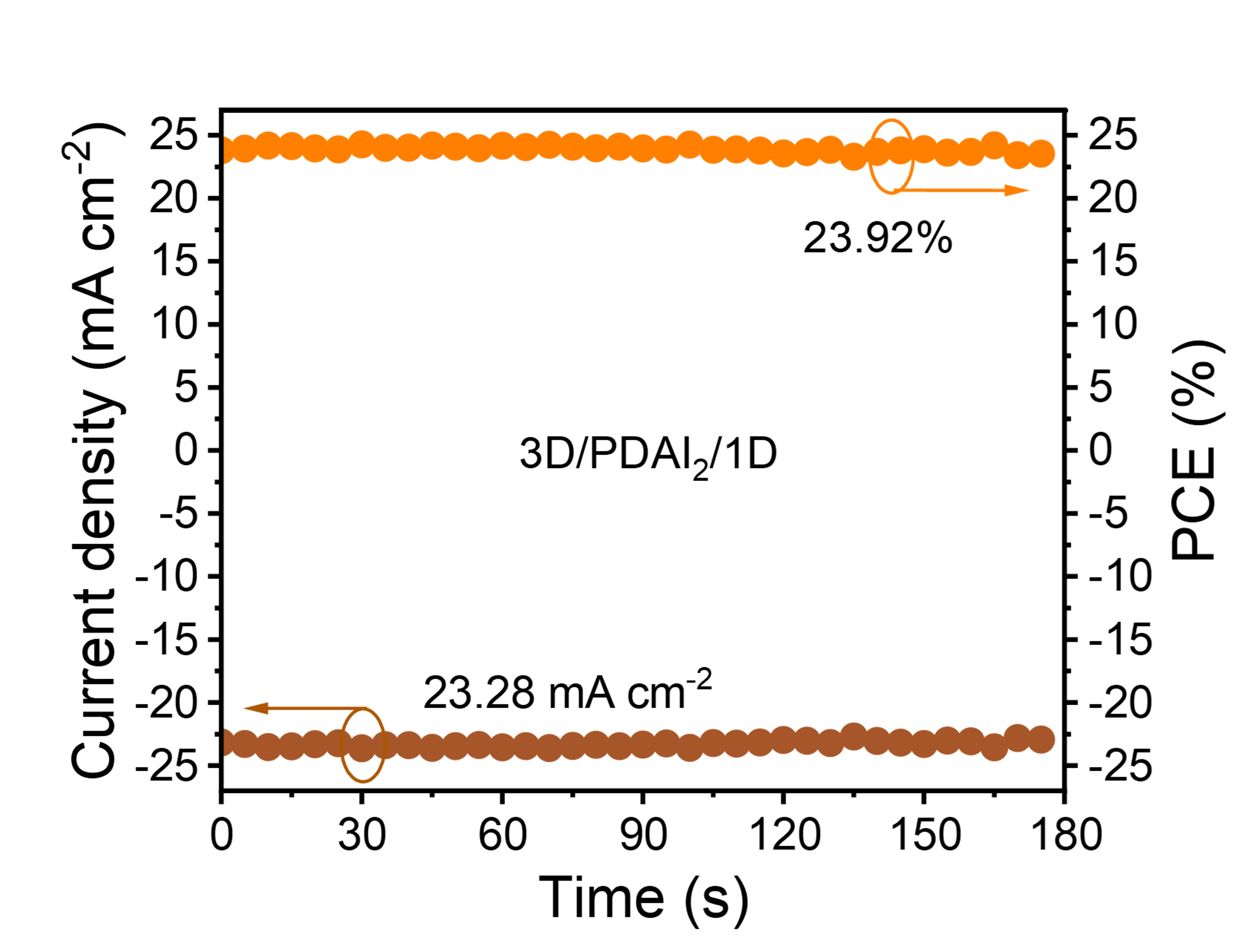


**Fig. S20** Stabilized power output of PSC with 3D/PDAI_2_/1D heterojunction of Figure 5b


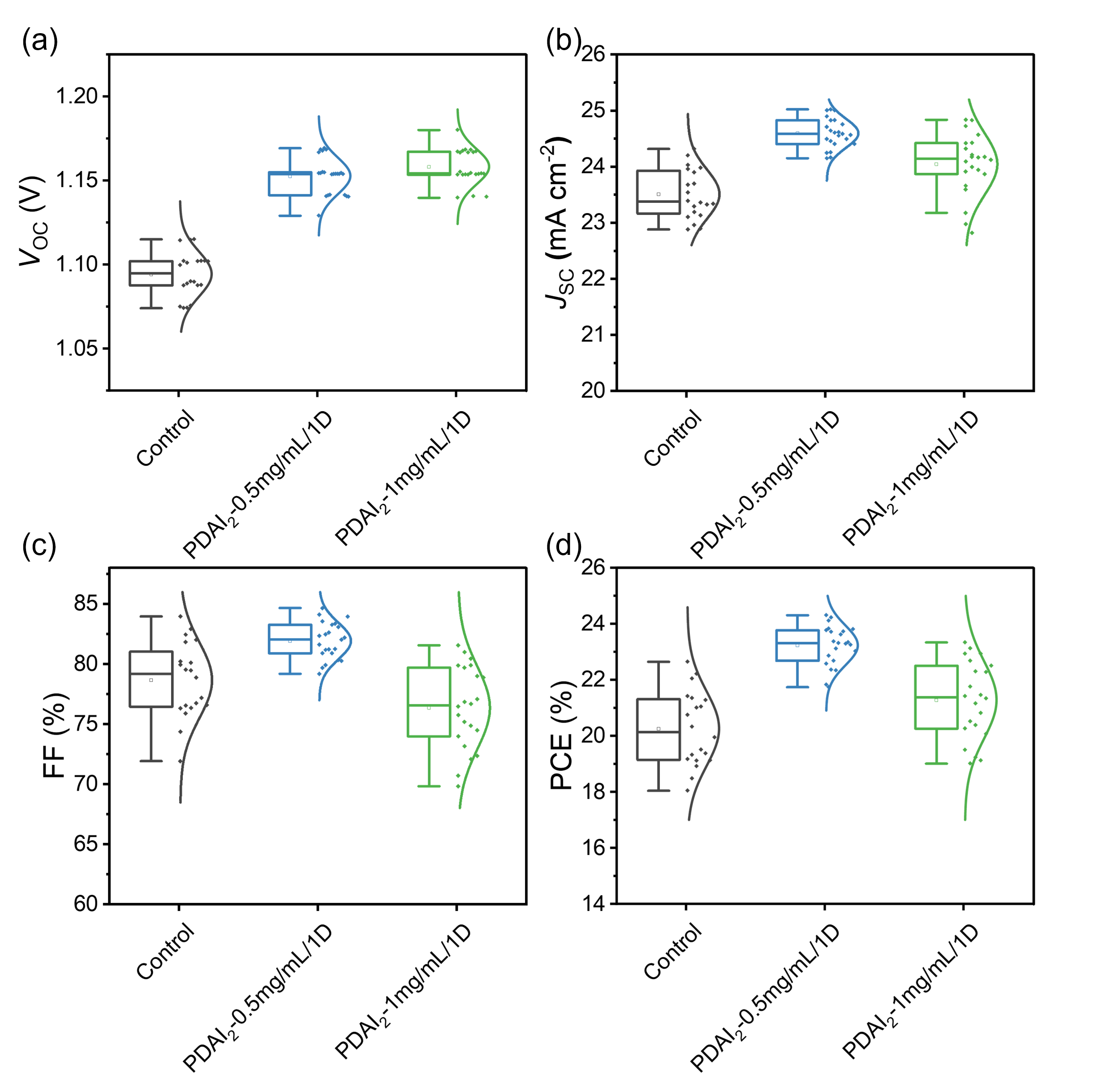


**Fig. S21** Box plots of **a** *V*_OC_, **b** *J*_SC_, **c** FF and **d** PCE of PSCs with different PDAI_2_ concentrations


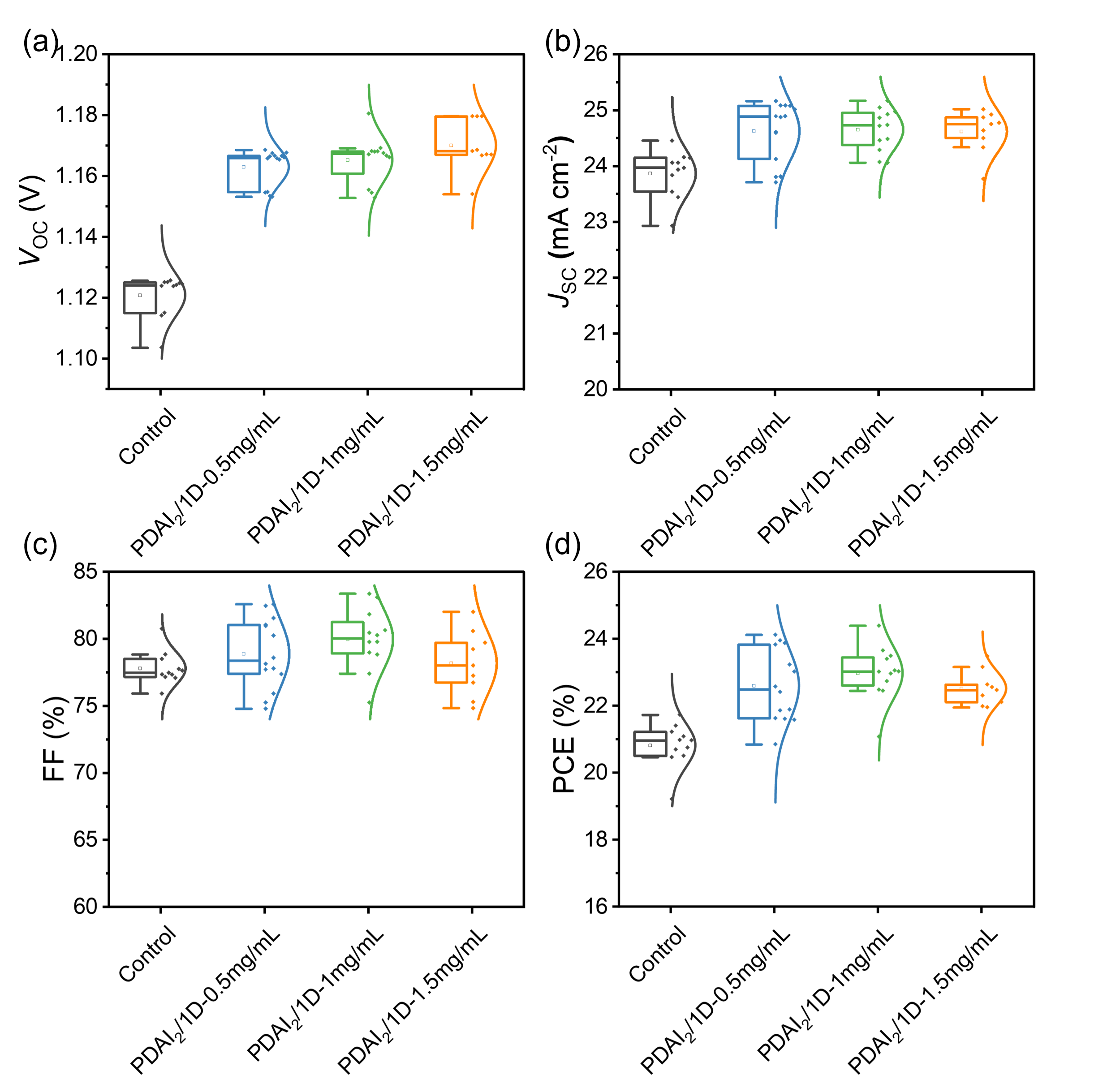


**Fig. S22** Box plots of **a** *V*_OC_, **b** *J*_SC_, **c FF** and **d** PCE of PSCs with different 4APyCl concentration

**Table S1**. Crystal data and structure refinement for (4APy)_2_PbI_4_ single crystal.

| Compound | (4APy)_2_PbI_4_ |
| --- | --- |
| Empirical formula | C_12_H_16_I_4_N_6_Pb |
| Formula weight | 959.10 |
| Temperature [K] | 226(7) |
| Crystal system | monoclinic |
| Space group (number) | $P2_{1}/n$ (14) |
| *a* [Å] | 4.0389(7) |
| *b* [Å] | 10.4245(19) |
| *c* [Å] | 21.865(4) |
| α [°] | 90 |
| β [°] | 94.015(17) |
| γ [°] | 90 |
| Volume [Å^3^] | 918.3(3) |
| *Z* | 2 |
| *F*(000) | 848 |

**Table S2** Atomic coordinates and *U*_eq_ [Å^2^] for (4APy)_2_PbI_4_ single crystal

| Atom | *x* | *y* | *z* | *U*_eq_ |
| --- | --- | --- | --- | --- |
| Pb1 | 0.500000 | 1.000000 | 0.500000 | 0.0300(10) |
| I2 | 0.0374(16) | 0.9918(4) | 0.5961(3) | 0.1249(17) |
| I1 | 0.5432(15) | 0.7285(6) | 0.49780(19) | 0.151(2) |
| C5 | 1.1212(16) | 0.6685(3) | 0.35225(18) | 0.026(3) |
| H5 | 1.123896 | 0.690961 | 0.393917 | 0.032 |
| N3 | 1.1839(19) | 0.7370(3) | 0.24835(12) | 0.032(3) |
| N2 | 0.8233(18) | 0.4796(10) | 0.42806(16) | 0.031(4) |
| H2A | 0.785914 | 0.421111 | 0.455121 | 0.037 |
| H2B | 0.785077 | 0.559884 | 0.435819 | 0.037 |
| C3 | 0.959(2) | 0.5257(4) | 0.26737(9) | 0.029(4) |
| H3 | 0.869556 | 0.448107 | 0.251653 | 0.035 |
| C6 | 1.230(2) | 0.7533(5) | 0.30844(10) | 0.033(4) |
| H6 | 1.343917 | 0.827365 | 0.322620 | 0.039 |
| C1 | 0.9341(15) | 0.4476(2) | 0.37704(13) | 0.031(4) |
| C4 | 1.0481(16) | 0.6234(3) | 0.2295(2) | 0.033(4) |
| H4 | 1.012000 | 0.610388 | 0.186976 | 0.039 |
| C2 | 1.0075(19) | 0.5480(3) | 0.33106(10) | 0.030(3) |
| N1 | 0.999(3) | 0.3286(3) | 0.3612(4) | 0.025(4) |
| H1A | 0.967095 | 0.265788 | 0.386323 | 0.030 |
| H1B | 1.074728 | 0.312874 | 0.325647 | 0.030 |

*U*_eq_ is defined as 1/3 of the trace of the orthogonalized *U_ij_* tensor.

**Table S3** Fitting parameters of the TRPL spectra for the PVK films with different surface treatment

| Sample | $\tau_{avg}$ (ns) | $\tau_{1}$ (ns) | A_1_ (%) | | $\tau_{2}$ (ns) | A_2_ (%) |
| --- | --- | --- | --- | --- | --- | --- |
| 3D | 783.96 | 97.99 | 19.89 | 804.70 | | 80.11 |
| 3D/1D | 2901.55 | 130.01 | 9.70 | 2914.83 | | 90.30 |
| 3D/PDAI_2_/1D | 3301.49 | 191.26 | 4.83 | 3310.60 | | 95.17 |

**Note S2**

TRPL decay curves are fitted by bi-exponential function:

$f\left( t \right)=A_{1}\exp\left( -t/\tau_{1} \right)+A_{2}\exp\left( -t/\tau_{2} \right)$

where $\tau_{1}$ and $\tau_{2}$ represent the lifetimes of charge carriers in distinct relaxation pathways, A_1_ and A_2_ are corresponding decay amplitudes. The average carrier lifetime $\tau_{avg}$ are calculated by function:

$\tau_{\mathrm{avg}}=(A_{1}\tau_{1}^{2}+A_{2}\tau_{2}^{2})/(A_{1}\tau_{1}+A_{2}\tau_{2})$

**Table S4** Photovoltaic parameters of Champion PSCs with 4 structures of Figure 5b

| Structure | Scan | *V*oc (V) | *J*sc *(*mA·cm^-2^) | FF (%) | PCE (%) | HI |
| --- | --- | --- | --- | --- | --- | --- |
| 3D | R | 1.102 | 24.3 | 82.9 | 22.2 | 0.05 |
|  | F | 1.100 | 23.9 | 79.9 | 21.0 |  |
| 3D/PDAI_2_ | R | 1.156 | 25.2 | 81.2 | 23.6 | 0.03 |
|  | F | 1.141 | 24.7 | 81.3 | 22.9 |  |
| 3D/1D | R | 1.115 | 25.3 | 84.0 | 23.7 | 0.03 |
|  | F | 1.116 | 25.0 | 82.4 | 23.0 |  |
| 3D/PDAI_2_/1D | R | 1.167 | 25.0 | 83.3 | 24.3 | 0.02 |
|  | F | 1.154 | 24.7 | 83.5 | 23.8 |  |

R represents Reverse scan, and F represents Forward scan.

**Table S5** Photovoltaic parameters PSCs of Figure 5f

| Structure | Scan | *V*oc (V) | *J*sc *(*mA·cm^-2^) | FF (%) | PCE (%) | HI |
| --- | --- | --- | --- | --- | --- | --- |
| 3D/PDAI_2_/1D | R | 1.170 | 26.0 | 84.9 | 25.8 | 0.01 |
|  | F | 1.170 | 25.9 | 84.4 | 25.6 |  |

R represents Reverse scan, and F represents Forward scan.

**Table S6** Summary of recently reported PSCs containing 3D/1D heterojunction (PCE>20%)

| 1D ligand | Device (Bulk/Interface) | PCE (%) | Refs. |
| --- | --- | --- | --- |
| DEAECl | n-i-p (Bulk) | 22.90 | [S1] |
| HABr | n-i-p(Bulk) | 21.20 | [S2] |
| TPI | p-i-n(Bulk) | 22.90 | [S3] |
| EMIMTFA | n-i-p(Bulk) | 22.14 | [S4] |
| TA-NI | n-i-p(Interface) | 23.84 | [S5] |
| Me3SI | n-i-p(Bulk) | 22.07 | [S6] |
| CBAH | n-i-p(Interface) | 21.95 | [S7] |
| p-PBAI2 | n-i-p(Interface) | 23.84 | [S8] |
| BnI | p-i-n(Bulk) | 21.17 | [S9] |
| Phen | n-i-p(Interface) | 23.30 | [S10] |
| BPy | n-i-p(Bulk) | 21.18 | [S11] |
| PAI | n-i-p(Interface) | 21.19 | [S12] |
| PyI | n-i-p(Interface) | 23.10 | [S13] |
| HDI | p-i-n(Interface) | 25.30 | [S14] |
| TBAAc | n-i-p(Interface) | 20.10 | [S15] |
| PyBr | n-i-p(Interface) | 23.74 | [S16] |
| TMA-TFSI | n-i-p(Interface) | 23.15 | [S17] |
| MTIm | p-i-n(Both) | 23.80 | [S18] |
| Benzimidazolium | n-i-p(Interface) | 24.43 | [S19] |
| CPMIMCl | n-i-p(Interface) | 24.13 | [S20] |
| PFACl | n-i-p(Interface) | 24.90 | [S21] |
| SMORCl | p-i-n(Interface) | 25.60 | [S22] |
| DMIMBF4 | n-i-p(Interface) | 24.75 | [S23] |
| 2ADPCl | n-i-p(Interface) | 24.55 | [S24] |
| C6I | n-i-p(Interface) | 23.11 | [S25] |
| BzMIMI | p-i-n(Interface) | 24.09 | [S26] |
| ABTI | p-i-n(Bulk) | 23.27 | [S27] |
| 4APyCl | p-i-n(Interface) | 25.80 | This work |

“Bulk/Interface” denotes the location where the 3D/1D heterojunction exists.

**Supplementary References**

1. T. Kong, H. Xie, Y. Zhang, J. Song, Y. Li et al., Perovskitoid-templated Formation of a 1D@3D perovskite structure toward highly efficient and stable perovskite solar cells. Adv. Energy Mater. **11**(34), 2101018 (2021). <https://doi.org/10.1002/aenm.202101018>
2. S. Yu, H. Liu, S. Wang, H. Zhu, X. Dong et al., Hydrazinium cation mixed FAPbI_3_^-^ based perovskite with 1D/3D hybrid dimension structure for efficient and stable solar cells. Chem. Eng. J. **403**, 125724 (2021). <https://doi.org/10.1016/j.cej.2020.125724>
3. H. Jiao, Z. Ni, Z. Shi, C. Fei, Y. Liu et al., Perovskite grain wrapping by converting interfaces and grain boundaries into robust and water-insoluble low-dimensional perovskites. Sci. Adv. **8**(48), eabq4524 (2022). <https://doi.org/10.1126/sciadv.abq4524>
4. N. Wei, Y. Chen, X. Wang, Y. Miao, Z. Qin et al., Multi-level passivation of MAPbI_3_ perovskite for efficient and stable photovoltaics. Adv. Funct. Mater. **32**(16), 2108944 (2022). <https://doi.org/10.1002/adfm.202108944>
5. Z. Chen, Q. Cheng, H. Chen, Y. Wu, J. Ding et al., Perovskite grain-boundary manipulation using room-temperature dynamic self-healing “ligaments” for developing highly stable flexible perovskite solar cells with 23.8% efficiency. Adv. Mater. **35**(18), 2300513 (2023). <https://doi.org/10.1002/adma.202300513>
6. C. Ge, J.-F. Lu, M. Singh, A. Ng, W. Yu et al., Mixed dimensional perovskites heterostructure for highly efficient and stable perovskite solar cells. Sol. RRL **6**(4), 2100879 (2022). <https://doi.org/10.1002/solr.202100879>
7. J. Wang, L. Liu, S. Chen, L. Qi, M. Zhao et al., Growth of 1D nanorod perovskite for surface passivation in FAPbI3 perovskite solar cells. Small **18**(3), 2104100 (2022). <https://doi.org/10.1002/smll.202104100>
8. J. Chen, Y. Yang, H. Dong, J. Li, X. Zhu et al., Highly efficient and stable perovskite solar cells enabled by low-dimensional perovskitoids. Sci. Adv. **8**(4), eabk2722 (2022). <https://doi.org/10.1126/sciadv.abk2722>
9. Y. Zhan, F. Yang, W. Chen, H. Chen, Y. Shen et al., Elastic lattice and excess charge carrier manipulation in 1D–3D perovskite solar cells for exceptionally long-term operational stability. Adv. Mater. **33**(48), 2105170 (2021). <https://doi.org/10.1002/adma.202105170>
10. Q. Chen, K. Deng, Y. Shen, L. Li, Stable one dimensional (1D)/three dimensional (3D) perovskite solar cell with an efficiency exceeding 23%. InfoMat **4**(5), e12303 (2022). <https://doi.org/10.1002/inf2.12303>
11. P. Liu, Y. Xian, W. Yuan, Y. Long, K. Liu et al., Lattice-matching structurally-stable 1D@3D perovskites toward highly efficient and stable solar cells. Adv. Energy Mater. **10**(17), 1903654 (2020). <https://doi.org/10.1002/aenm.201903654>
12. N. Yang, C. Zhu, Y. Chen, H. Zai, C. Wang et al., An *in situ* cross-linked 1D/3D perovskite heterostructure improves the stability of hybrid perovskite solar cells for over 3000 h operation. Energy Environ. Sci. **13**(11), 4344–4352 (2020). <https://doi.org/10.1039/D0EE01736A>
13. H.B. Lee, A. Mohamed, N. Kumar, N.H. Zain Karimy, V.V. Satale et al., Low-cost, scalable fabrication of multi-dimensional perovskite solar cells and modules assisted by mechanical scribing. Small Methods **9**(1), 2400850 (2025). <https://doi.org/10.1002/smtd.202400850>
14. Y. Zhao, Y.-C. Ye, X.-M. Chen, J.-G. Wang, L.-J. Zhang et al., Surface impurity healing by the formation of 1D/3D perovskite heterojunctions for inverted perovskite solar cells. ACS Sustainable Chem. Eng. **13**(32), 13148–13156 (2025). <https://doi.org/10.1021/acssuschemeng.5c06468>
15. A. Mei, X. Peng, X. Li, G. Zhang, S. Lin et al., Anions regulation of 1D perovskite intrusion-behavior for efficient and stable perovskite solar cells. ACS Appl. Mater. Interfaces **16**(24), 31209–31217 (2024). <https://doi.org/10.1021/acsami.4c06087>
16. J. Cha, C. Beom Lee, S. Min Park, D. Baek, S. Kim et al., Lattice-matched in-situ-formed 1D perovskite phase in Multi-dimensional solar cells achieving high phase stability and favorable energy landscape. Chem. Eng. J. **484**, 149280 (2024). <https://doi.org/10.1016/j.cej.2024.149280>
17. S.-K. Jung, K. Park, J.-H. Lee, S.-G. Choi, J.S. Choi et al., Exploiting an interfacial reaction in perovskite solar cells: quaternary alkylammonium ionic salt dopants for spiro-OMeTAD. Adv. Energy Mater. **15**(2), 2402144 (2025). <https://doi.org/10.1002/aenm.202402144>
18. C. Zhou, F. Wang, X. Ai, Y. Liu, Y. Han et al., Dual interfacial modification with 1D perovskite for self-assembled monolayer based inverted perovskite solar cells. Nano Energy **128**, 109811 (2024). <https://doi.org/10.1016/j.nanoen.2024.109811>
19. R. Chen, H. Shen, Q. Chang, Z. Tang, S. Nie et al., Conformal imidazolium 1D perovskite capping layer stabilized 3D perovskite films for efficient solar modules. Adv. Sci. **9**(36), 2204017 (2022). <https://doi.org/10.1002/advs.202204017>
20. F. Wang, D. Duan, K. Zhou, Y.Z.B. Xue, X. Liang et al., Ionic liquid engineering enabled in-plane orientated 1D perovskite nanorods for efficient mixed-dimensional perovskite photovoltaics. InfoMat **5**(8), e12459 (2023). <https://doi.org/10.1002/inf2.12459>
21. F. Ye, T. Tian, J. Su, R. Jiang, J. Li et al., Tailoring low-dimensional perovskites passivation for efficient two-step-processed FAPbI3 solar cells and modules. Adv. Energy Mater. **14**(4), 2302775 (2024). <https://doi.org/10.1002/aenm.202302775>
22. S. Li, H. Gu, A. Zhu, J. Guo, C. Xi et al., Anion-cation synergistic regulation of low-dimensional perovskite passivation layer for perovskite solar cells. Adv. Mater. **37**(28), 2500988 (2025). <https://doi.org/10.1002/adma.202500988>
23. F. Wang, K. Zhou, C. Zhou, X. Liang, T. Wang et al., Ionic liquid-induced 1D perovskite: exploring 1D perovskite structure to 1D/3D heterojunction-based photovoltaics. Adv. Energy Mater. **14**(23), 2400021 (2024). <https://doi.org/10.1002/aenm.202400021>
24. X. Zhou, X. Liang, F. Wang, H. Sun, Q. Zhu et al., Pyridine substitution strategy for one-dimensional perovskite: Toward efficient and stable mixed-dimensional photovoltaics. Chem. Eng. J. **493**, 152539 (2024). <https://doi.org/10.1016/j.cej.2024.152539>
25. Y. Hu, L. Gao, H. Su, X. Du, N. Yuan et al., Amino acid-based low-dimensional management for enhanced perovskite solar cells. Sol. RRL **6**(7), 2200168 (2022). <https://doi.org/10.1002/solr.202200168>
26. D. Wang, J. Chen, P. Zhu, Y. Qiao, H. Hu et al., Binary microcrystal additives enabled antisolvent-free perovskite solar cells with high efficiency and stability. Adv. Energy Mater. **13**(7), 2203649 (2023). <https://doi.org/10.1002/aenm.202203649>
27. Y. Zhang, C. Li, E. Bi, T. Wang, P. Zhang et al., Efficient inverted perovskite solar cells with a low-dimensional halide/perovskite heterostructure. Adv. Energy Mater. **12**(48), 2202191 (2022). <https://doi.org/10.1002/aenm.202202191>
